# Acyl-Protein Thioesterase 2 Catalizes the Deacylation of Peripheral Membrane-Associated GAP-43

**DOI:** 10.1371/journal.pone.0015045

**Published:** 2010-11-30

**Authors:** Vanesa M. Tomatis, Alejandra Trenchi, Guillermo A. Gomez, Jose L. Daniotti

**Affiliations:** Centro de Investigaciones en Química Biológica de Córdoba (CIQUIBIC, UNC-CONICET), Departamento de Química Biológica, Facultad de Ciencias Químicas, Universidad Nacional de Córdoba, Córdoba, Argentina; University of South Florida College of Medicine, United States of America

## Abstract

An acylation/deacylation cycle is necessary to maintain the steady-state subcellular distribution and biological activity of S-acylated peripheral proteins. Despite the progress that has been made in identifying and characterizing palmitoyltransferases (PATs), much less is known about the thioesterases involved in protein deacylation. In this work, we investigated the deacylation of growth-associated protein-43 (GAP-43), a dually acylated protein at cysteine residues 3 and 4. Using fluorescent fusion constructs, we measured in vivo the rate of deacylation of GAP-43 and its single acylated mutants in Chinese hamster ovary (CHO)-K1 and human HeLa cells. Biochemical and live cell imaging experiments demonstrated that single acylated mutants were completely deacylated with similar kinetic in both cell types. By RT-PCR we observed that acyl-protein thioesterase 1 (APT-1), the only bona fide thioesterase shown to mediate deacylation in vivo, is expressed in HeLa cells, but not in CHO-K1 cells. However, APT-1 overexpression neither increased the deacylation rate of single acylated GAP-43 nor affected the steady-state subcellular distribution of dually acylated GAP-43 both in CHO-K1 and HeLa cells, indicating that GAP-43 deacylation is not mediated by APT-1. Accordingly, we performed a bioinformatic search to identify putative candidates with acyl-protein thioesterase activity. Among several candidates, we found that APT-2 is expressed both in CHO-K1 and HeLa cells and its overexpression increased the deacylation rate of single acylated GAP-43 and affected the steady-state localization of diacylated GAP-43 and H-Ras. Thus, the results demonstrate that APT-2 is the protein thioesterase involved in the acylation/deacylation cycle operating in GAP-43 subcellular distribution.

## Introduction

Fatty-acylated peripheral proteins such as members of the small G-protein Ras family, heterotrimeric G-proteins, the neuronal proteins PSD-95 and growth-associated protein-43 (GAP-43) [1]–[4] are synthesized in the cytosol and posttranlationally modified by different lipid moieties [5]–[9]. These lipid modifications govern their membrane association and membrane subdomain segregation as well as their trafficking, function and stability [8], [10], [11].

Among all posttranslational lipid modification of proteins, including isoprenylation and myristoylation, the addition of fatty acid to the sulfhydryl group of a cysteine to form a thioester bond (S-acylation, often referred to as palmitoylation) is the only readily reversible linkage having a much shorter half-life than that of the protein [12]–[15]. Consequently, S-acylation can operate as a switch regulating not only protein-membrane binding affinity and segregation but also modulating its biological activities [16]–[18]. S-acylation is catalyzed by protein acyltransferases (PATs) while deacylation by acyl-protein thioesterases (APTs), and continuous cycles of de- and reacylation reactions accounts for the specific subcellular distribution of peripheral proteins as the small GTPases H- and N-Ras [19]–[22].

PATs have been identified both in yeast and mammalian [18]. These proteins share a common zinc finger-like sequence, containing a cysteine-rich domain with an aspartate-histidine-histidine-cysteine (DHHC) motif, which mediates the PAT activity. Although S-acylation was reported to occur in several membrane compartments [22]–[24] and with apparent substrate selectivity, a recent work from Bastiaens and coworker demonstrated that S-acylation of semisynthetic substrates is detectable only in the Golgi complex and, that substrate specificity is not essential for the reacylation step [21]. Despite the progress that has been made in identifying and characterizing PATs much less is known about the thioesterases that deacylate proteins. So far, only two APT has been described: palmitoyl-protein thioesterase 1 (PPT1) and acyl-protein thioesterase 1 (APT-1). PPT1 was discovered based on its ability to deacylate H-Ras [25]. Further research revealed that PPT1 is a lysosomal enzyme involved in protein degradation [26] discarding the possibility to play a role in deacylation of cytoplasmic proteins. APT-1, originally isolated from rat liver as a lysophospholipase [27], is a cytosolic protein with a widespread tissue distribution. Several proteins have been identified as APT-1 substrates, like heterotrimeric G protein α subunits, endothelial nitric-oxide synthase, SNAP-23 and H-Ras as well as viral proteins [18]. In opposite to acylation, the deacylation step seems to occurs everywhere in the cell and no specific consensus sequence or substrate specificity has been described for this enzymatic reaction so far [21].

In this work, we demonstrated for the first time that lysophospholipase II or APT-2 is a cytosolic protein thioesterase involved in GAP-43 deacylation. GAP-43 was early identified as a functional growth cone marker participating in the mechanisms of axonal outgrowth and regeneration [28]–[31]. Then, it was also identified in peripheral and central glia cells and developing muscle cells [32], [33], which points to a fundamental role for GAP-43 in cellular processes. After synthesis in the cytosol, GAP-43 binds to trans Golgi network (TGN) membranes through a process that requires acylation of cysteine residues at positions 3 and 4 [34], [35]. This posttranslational modification is necessary for GAP-43 inclusion into exocytic and endocytic vesicular carriers.

Although several groups have described the molecular determinants involved in the polarized sorting of GAP-43 in neural cells [34], [36]–[39], it is still unclear how this subcellular distribution is maintained. An attractive hypothesis is that cycles of deacylation/acylation controls GAP-43 localization and sorting [4], [40], [41]. In this sense, it has been recently described that the fast kinetics and topography of the acylation cycle allows the continuous redirection of mislocalized proteins via the post-Golgi sorting apparatus [21]. In addition, the presence of PATs at the plasma membrane has been also demonstrated [3], [34].

Using in vivo confocal fluorescence microscopy, biochemical analyses and bioinformatic approaches, we characterized the deacylation kinetics of GAP-43 and identified the thioesterase involved in this process. It was found that GAP-43 is deacylated in vivo when expressed in Chinese hamster ovary (CHO)-K1 and human HeLa cells through a mechanism mediated by APT-2, but not by APT-1. Inhibition of deacylation caused GAP-43 to accumulate in perinuclear structures while APT-2 overexpression increased the cytoplasmic pool of GAP-43. Additionally, it was also demonstrated that APT-2 overexpression affected the steady-state subcellular localization of acylated H-Ras. Thus, the results demonstrate that APT-2 is the protein thioesterase involved in the acylation/deacylation cycle operating in GAP-43 subcellular distribution.

## Results

### The Full-Length and N-terminal Domains of GAP-43 are Deacylated In Vivo with Relatively Slow Kinetics

**Figure 1:**
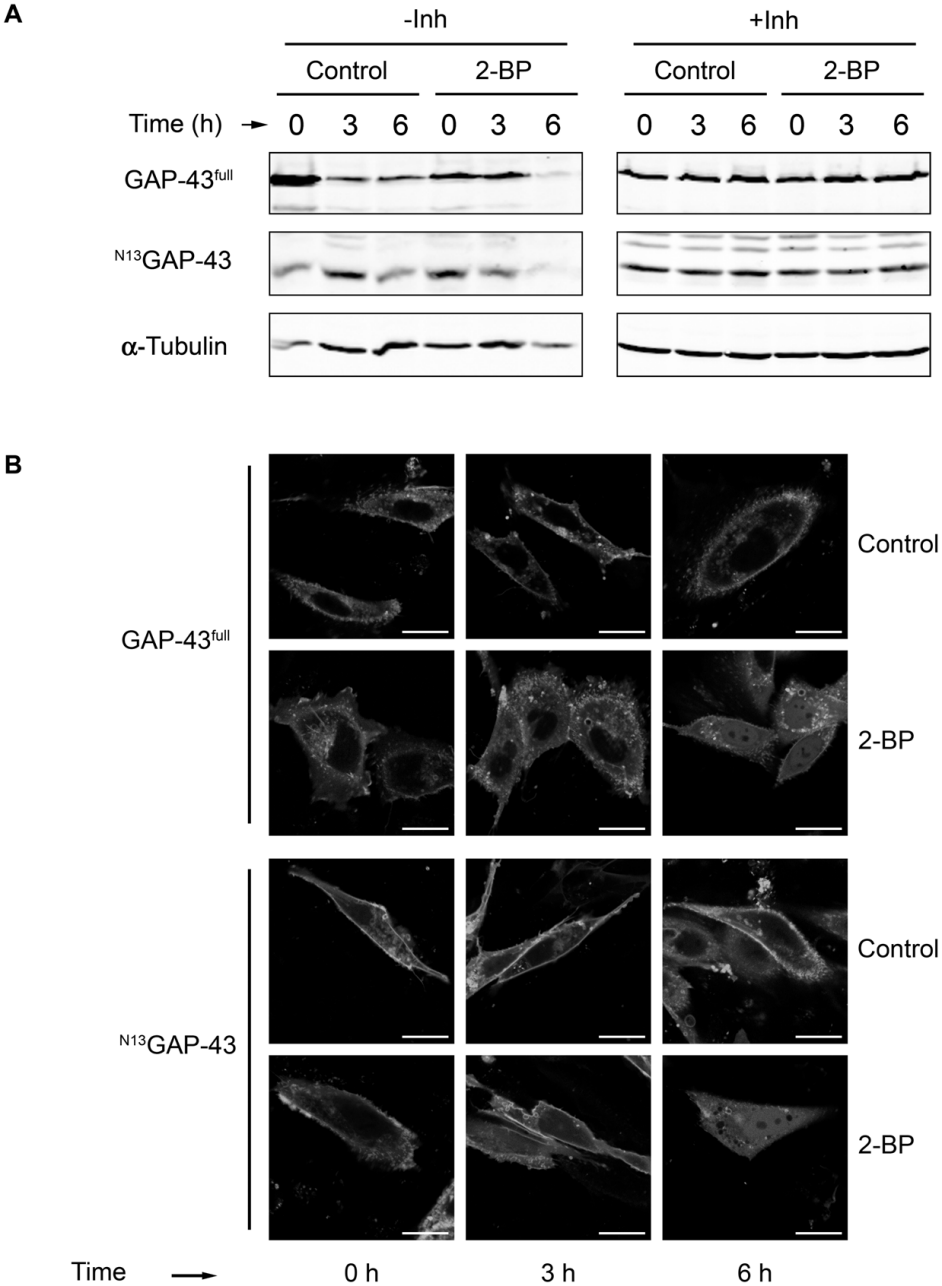
GAP-43 deacylation in CHO-K1 cells. **A**) 24 h after transfection with plasmids for GAP-43^full^-YFP or ^N13^GAP-43-YFP, CHO-K1 cells were incubated with 50 µM 2-BP (2-BP) or vehicle (Control) in the presence (+Inh) or absence (-Inh) of protein degradation inhibitors for 0, 3 and 6 h. At each time point, cells were lysed and homogenates Western blotted with an antibody to GFP. The lower panels show Western blot with antibody to α-tubulin. **B**) 60 h after transfection, CHO-K1 cells expressing GAP-43^full^-YFP or ^N13^GAP-43-YFP were treated with 50 µM 2-BP (2-BP) or vehicle (Control) for 0, 3 and 6 h in presence of CHX and protein degradation inhibitors. At each time point the subcellular distribution was analyzed by live cell confocal fluorescent microscopy. Scale bars: 5 µm.

Previously, we characterized the acylation, membrane association and intracellular trafficking of full-length (GAP-43^full^) and the acylation motif (MLCCMRRTKQVEK, ^N13^GAP-43) of GAP-43 fused to spectral variants of green fluorescent protein (GFP) [cyan fluorescent protein (CFP) or yellow fluorescent protein (YFP)] [34], [42]. We demonstrated that both constructs are acylated at the TGN and subsequently transported to the plasma membrane by vesicular carriers. At the plasma membrane, these proteins are endocytosed and directed to the recycling endosomes (RE) in an Arf6-dependent manner. Thus, this intracellular itinerary determines the presence of GAP-43 at the TGN, plasma membrane and RE at steady-state conditions [34], [42]. In experiments carried out to investigate GAP-43 deacylation in CHO-K1 cells, it was observed that PATs inhibition with 100 µM 2-bromopalmitate (2-BP) completely abolished acylation of newly synthesized GAP-43 and therefore its binding to the membranes. However, at steady-state conditions 2-BP treatment did not modify the acylation state and membrane binding properties of GAP-43 [34], strongly suggesting that membrane-associated GAP-43 is not being deacylated. However, it should be also considered that deacylated GAP-43 could be a preferred substrate for proteasome degradation [43], [44], avoiding cytosolic accumulation, and/or that 2-BP not only inhibits PATs but also APT activity similarly to that observed using APT-1 inhibitors [45]. To assess these alternatives, cells expressing GAP-43 at steady-state conditions were treated with 2-BP and cycloheximide (CHX) in the presence (+Inh) or absence (-Inh) of proteasomal and lysosomal inhibitors during 0, 3 and 6 h (Fig. 1). As shown in Figure 1A, a significant degradation of GAP-43 was observed along the time in the absence of inhibitors. Contrary and supporting the previous assumption, GAP-43 proteolysis was abolished when cells were incubated with 2-BP and CHX in the presence of protein degradation inhibitors.

Next, we investigated the in vivo deacylation kinetic of GAP-43^full^ and ^N13^GAP-43 (Fig. 1B). Cells were treated with 2-BP during 0, 3 and 6 h and the subcellular distribution analyzed by in vivo confocal fluorescent microscopy. CHX and inhibitors of protein degradation were incorporated to the culture medium 1 h before 2-BP addition. All experiments were performed using 50 µM 2-BP to inhibit PATs without affecting APT activity (Tomatis, Gomez and Daniotti, unpublished results) [34]. At 0, 3 and 6 h, both ^N13^GAP-43 and GAP-43^full^ localized at the plasma membrane and perinuclear structures in control cells, as previously reported [34]. In contrast, in 2-BP-treated cells there was a significant amount of soluble, nonacylated GAP-43 protein at 3 and 6 h, being this more evident for GAP-43^full^ than ^N13^GAP-43. We discarded accumulation of newly synthesized GAP-43 since CHX was present during all the experiment. Altogether, these results support the existence of an acylation/deacylation cycle which operates in the membrane binding and subcellular distribution of GAP-43. Additionally, the observed deacylation kinetic for GAP-43^full^ and ^N13^GAP-43 was found to be comparable to that previously described for H-Ras [19].

### Single Acylated Mutant of ^N13^GAP-43 is Deacylated In Vivo Faster than its Double Acylated Counterpart

Diacylated GAP-43 requires a double deacylation event for its membrane dissociation. To investigate single deacylation events occurring on GAP-43 and to set a more direct method for deacylation readout, we decided to carry out our experiments using single acylated mutants of GAP-43. Importantly, it should also be mentioned that at steady-state conditions the monopalmitoylated fraction of GAP-43 represent the 60% of the total GAP-43 protein, indicating the importance of the monoacylated specie in GAP-43 function [46].

**Figure 2:**
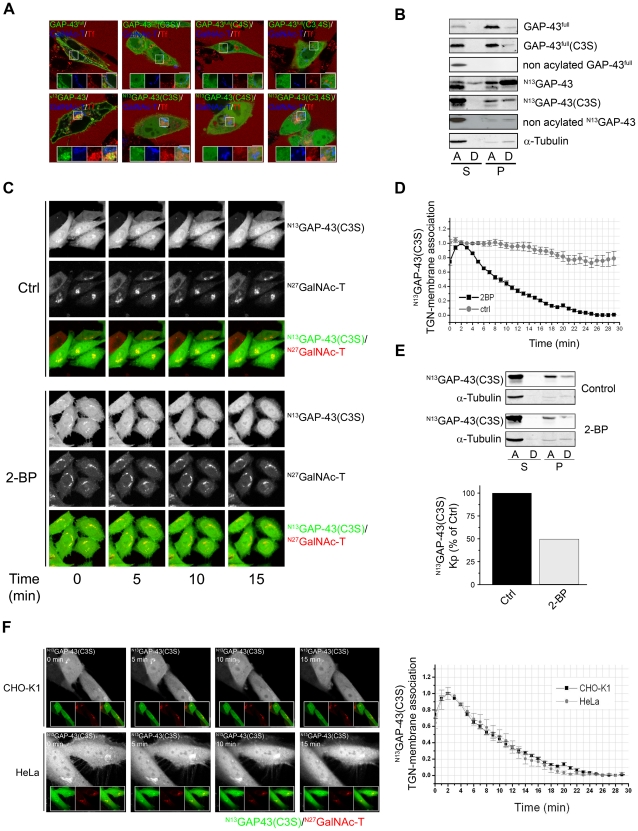
Deacylation kinetic of single acylated ^N13^GAP-43(C3S). **A**) 60 h after coexpressing GAP-43^full^-YFP (GAP-43^full^), ^N13^GAP-43-YFP (^N13^GAP-43), their S-acylation mutants (C3S, C4S or C3,4S) and ^N27^GalNAc-T-CFP (GalNAc-T), CHO-K1 cells were incubated with Alexa Fluor 647-Tf (Tf) to label the RE and analyzed by live cell confocal fluorescent microscopy. The fluorescence signals from YFP, CFP and Alexa Fluor 647 were pseudocolored green, blue and red, respectively. Each panel shows merged images from the three acquired channels. The insets in each panel show details of the boxed area at a higher magnification with the corresponding pseudocoloured signal from each channel. Scale bars: 5 µm. **B**) CHO-K1 cells transiently expressing GAP-43^full^-YFP (GAP-43^full^), GAP-43^full^(C3S)-YFP [GAP-43^full^(C3S)], ^N13^GAP-43-YFP (^N13^GAP-43) or ^N13^GAP-43(C3S)-YFP [^N13^GAP-43(C3S)] were lysed, ultracentrifuged and the S (soluble) and P (pellet) fraction isolated. Buffer containing 1% v/v Triton X-114 was added to samples and phase separation was induced at 37°C. Proteins from the A (aqueous) and D (detergent) phases were Western blotted with an antibody to GFP. To obtain nonacylated GAP-43^full^ and ^N13^GAP-43, CHO-K1 cells were transfected with the corresponding plasmids in presence of CHX. 30 min before CHX withdrawal, cells were incubated with 50 µM 2-BP until 3 h after CHX withdrawal. S and P fractions were isolated and analyzed by Western blot as explained above. The lower panels show the Western blot using antibody to α-tubulin. **C**) 60 h after transient transfection, CHO-K1 cells coexpressing ^N13^GAP-43(C3S) and ^N27^GalNAc-T were treated with 50 µM 2-BP (2-BP, lower panels) or vehicle (Ctrl, upper panels) in presence of CHX and protein degradation inhibitors during 30 min before imaging and during all the experiment. GAP-43 subcellular distribution was analyzed by live cell confocal microscopy. Panels show YFP (pseudocolored gray) and CFP (pseudocolored gray), and merge images from YFP (pseudocolored green) and CFP (pseudocolored red) signals at 0, 5, 10 and 15 minutes after 2-BP or vehicle addition. Scale bars: 5 µm. **D**) Quantification of images showed in 2**C** (see Materials and Methods). **E**) Upper, 24 h after transfection, CHO-K1 cells transiently expressing ^N13^GAP-43(C3S)-YFP [^N13^GAP-43(C3S)] were lysed, ultracentrifuged and the S and P fraction were recovered. Buffer containing 1% v/v Triton X-114 was added to samples and phase separation was induced at 37°C. Proteins from the A and D phases were Western blotted with an antibody to GFP and α-tubulin. Lower, quantification (as described in Materials and Methods) of Western blot showed in upper panels. **F**) Left, 60 h after transient transfection, CHO-K1 and HeLa cells coexpressing ^N13^GAP-43(C3S)-YFP and ^N27^GalNAc-T-CFP were treated with 50 µM 2-BP (2-BP, lower panels) or vehicle (Control, upper panels) in presence of protein degradation inhibitors during 30 min. GAP-43 subcellular distribution was analyzed by live cell confocal microscopy. Each panel shows image from YPF (pseudocoloured gray) channel at 0, 5, 10 and 15 min from the beginning of the experiment. The insets in each panel show (from left to right): YFP fluorescence (pseudocolored green), CFP fluorescence (pseudocolored red) fluorescence and the merge of both fluorescence signals. Scale bars: 5 µm. Right, quantification of images showed in left panels (see Materials and Methods).

We generated single and double cysteine mutants at the acylation motif of ^N13^GAP-43 [^N13^GAP-43(C3S), ^N13^GAP-43(C4S), ^N13^GAP-43(C3,4S)] and GAP-43^full^ [GAP-43^full^(C3S), GAP-43^full^(C4S), GAP-43^full^(C3,4S)] which were then characterized by in vivo confocal microscopy and biochemical analyses. As previously observed, GAP-43^full^ and ^N13^GAP-43 localized, at steady-state, at the RE, plasma membrane and TGN (Fig. 2A). The point mutation at Cys^3^ in ^N13^GAP-43 caused an accumulation at the cytosol and TGN, and disrupted RE association, but did not affect their plasma membrane association. In contrast, the point mutation at Cys^3^ in GAP-43^full^ disrupted the association with the plasma membrane, drastically reduced their TGN membrane association, and resulted in an accumulation at the cytosol. Double mutants at Cys^3^ and Cys^4^ of GAP-43^full^ and ^N13^GAP-43 were not able to associate to any membranes and occurred diffusely throughout the cells, characteristic of cytosolic proteins (Fig. 2A).

The membrane association and the extent of posttranslational modification of these constructs was further characterized by ultracentrifugation and Triton X-114 (TX-114) partition assays (Fig. 2B) [47]. More than 70% of GAP-43^full^ and ^N13^GAP-43 were found to be associated with the pellet (P) fraction after ultracentrifugation. 20% and 70% of P fraction-associated GAP-43^full^ and ^N13^GAP-43, respectively, were found enriched in the TX-114 detergent (D) phase, reflecting a higher hydrophobic character for ^N13^GAP-43. In contrast, a drastic reduction in membrane association (<5% in P fraction) of newly synthesized GAP-43 was observed when cells were incubated with the PAT inhibitor 2-BP (see nonacylated ^N13^GAP-43 and nonacylated GAP-43^full^ in Fig. 2B). As additional control, we demonstrated that soluble α-tubulin is mostly associated to the A (aqueous) phase of the soluble fraction, in agreement to the biochemical properties of this protein. 54% of GAP-43^full^(C3S) and 35% of ^N13^GAP-43(C3S) were found to be associated with the P fraction. Additionally, 20% and 43% of P fraction-associated GAP-43^full^(C3S) and ^N13^GAP-43(C3S), respectively, were found enriched in the D phase, reflecting a higher hydrophobic character for ^N13^GAP-43(C3S) (Fig. 2B). Altogether, these results demonstrate that acylation is necessary for GAP-43 membrane binding and that ^N13^GAP-43(C3S) is the single acylation mutant having the highest proportion of acylation and, consequently, membrane association.

Next, we evaluated the deacylation kinetics of ^N13^GAP-43(C3S) in order to investigate single deacylation events occurring on GAP-43. CHO-K1 cells transiently coexpressing ^N13^GAP-43(C3S) and ^N27^GalNAc-T (TGN marker) were treated with 50 µM 2-BP (2-BP) or dimethylsulfoxide (DMSO, Control) and GAP-43 subcellular distribution monitored at different times by live cell confocal fluorescent microscopy (Fig. 2C). CHX and protein degradation inhibitors were incorporated to the culture medium 1 h before and during 2-BP treatment. In control cells, the amount of TGN-membrane association of GAP-43(C3S) did not significantly change over time. In opposite, TGN-associated GAP-43 significantly decreased over time in 2-BP treated cells with a half-life of 6 min (Fig. 2C and D) clearly much faster than its wild-type diacylated counterpart (see Fig. 1). The observed decrease of the TGN-membrane association of GAP-43(C3S) in 2-BP conditions was not attributed to TGN membrane redistribution, since the TGN marker ^N27^GalNAc-T was not affected under these experimental conditions, nor to an increase in TGN to plasma membrane vesicular transport of ^N13^GAP-43(C3S) since all experiments were performed at 20°C, a condition which drastically decreases this process [34].

To support the in vivo microscopy experiments, membrane association and extent of posttranslational modification of ^N13^GAP-43(C3S) were determined at the same experimental conditions by subcellular fractionation and TX-114 partition assay. It was found that 2-BP, when compared to control conditions, caused a significant decrease of membrane bounded-^N13^GAP-43(C3S) (Fig. 2E). A similar behavior was observed for GAP-43^full^(C3S) (Fig. S1). Overall, these biochemical results are in close agreement with those obtained by live cell confocal microscopy analysis. To discard that the observed GAP-43 deacylation process could be a cell type-specific phenomenon, then, we extended our analysis using human HeLa cells and found that ^N13^GAP-43(C3S) is deacylated with a similar kinetics both in HeLa and CHO-K1 cells (Fig. 2F), precluding the possibility that GAP-43 deacylation is a cell type specific process.

These results demonstrate that ^N13^GAP-43(C3S) is deacylated in vivo and that GAP-43 deacylation occurs at the TGN, besides the plasma membrane, revealing the importance of an acylation/deacylation cycle operating in GAP-43 subcellular distribution [17], [19], [22], [48], [49].

### GAP-43 Deacylation in CHO-K1 Cells is APT-1 Independent

Up to now, results indicate that ^N13^GAP-43(C3S) is deacylated with similar kinetic when it is expressed in CHO-K1 and HeLa cells. APT-1 is the unique described cytosolic protein thioesterase able to catalyze the deacylation of several substrates including H-Ras and G alpha subunit proteins [13], [18], [50]. However, there is no information about the role of this enzyme on GAP-43 deacylation.

**Figure 3:**
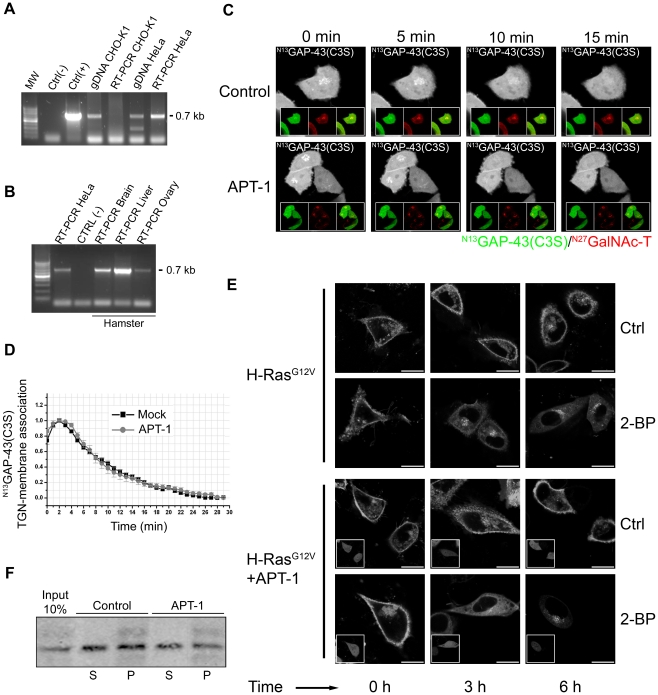
GAP-43 deacylation is not mediated by APT-1. **A**) PCR analysis of APT-1 expression using specific primers and the following templates: the reaction mixture [ctrl (-)]; a plasmid containing the cDNA coding APT-1 [ctrl (+)]; genomic DNA from CHO-K1 cells (gDNA CHO-K1); first-strand cDNA obtained from RT of CHO-K1 cells purified mRNA (RT-PCR CHO-K1 cells); genomic DNA from HeLa cells (gDNA HeLa) and first-strand cDNA obtained from RT of HeLa cells purified mRNA (RT-PCR HeLa cells). Genomic DNA and mRNA were extracted from CHO-K1 and HeLa cells. **B**) PCR analysis of APT-1 expression using as template: first-strand cDNA obtained from RT of HeLa cells purified mRNA [ctrl (+)]; the reaction mixture [ctrl (-)] and first-strand cDNA obtained from mRNA of hamster brain (RT-PCR Brain), liver (RT-PCR Liver) and ovary (RT-PCR Ovary). **C**) 60 h after transient transfection, CHO-K1 cells expressing ^N13^GAP-43(C3S) alone (Control, upper panels) or coexpressing ^N13^GAP-43(C3S) and APT-1(APT-1, lower panels) were treated with 50 µM 2-BP. CHX and protein degradation inhibitors were added 30 min before imaging and maintained in the culture media until the end of each experiment. GAP-43 subcellular distribution was monitored by live cell confocal microscopy. Each panel shows image from YPF fluorescence (pseudocoloured gray) at 0, 5, 10 and 15 min from 2-BP addition. The insets in each panel show (left to right): YFP fluorescence (pseudocoloured green), CFP fluorescence (pseudocoloured red) and the merge of both fluorescence signals. **D**) Quantification [^N13^GAP-43(C3S) associated to TGN membranes] of images showed in panels **C** (see Materials and Methods). **E**) 60 h after transient transfection, CHO-K1 cells expressing H-RasG12V (upper panels) or coexpressing H-RasG12V and APT-1 (lower panels) were treated with 50 µM 2-BP (2-BP) or vehicle (Ctrl) in presence of CHX and protein degradation inhibitors for 0, 3 and 6 h and the subcellular distribution analyzed by live cell confocal microscopy. Each panel shows image from YFP fluorescence (pseudocoloured gray). The insets show the cells expressing APT-1 (cherry fluorescence). **F**) S fraction from CHO-K1 cells transiently expressing ^N13^GAP-43(C3S) was incubated with P fraction from nontransfected CHO-K1 cells during 1 h at 4°C. Then, the P fraction was isolated by ultracentrifugation and incubated in the presence (APT-1) or the absence (Control) of recombinant APT-1. Then, S and P fractions were separated by ultracentrifugation and proteins from each fraction Western blotted using an antibody to GFP. Scale bars: 5 µm.

To investigate whether APT-1 deacylates GAP-43, we first analyzed mRNA expression of APT-1 both in CHO-K1 and HeLa cells by reverse transcriptase-polimerase reaction chain (RT-PCR). The results showed that a PCR product of the expected size was obtained using mRNAs from HeLa cells as template (Fig. 3A). The same result was also obtained using mRNA from African green monkey kidney fibroblast cells (COS-7 line) (Fig. S2). In opposite, no amplification of any product was observed using mRNA purified from CHO-K1 cells. Primer specificity was validated by APT-1 cDNA amplification using genomic DNA from CHO-K1 cells and mRNA purified from hamster brain, liver and ovary tissues (Fig. 3A and B). The identity of all PCR products was confirmed by DNA sequencing and results showed identical amino acid sequence between human and hamster APT-1 (results not shown).

Thus, results demonstrated that APT-1 is not expressed in CHO-K1 cells. To discard the possibility that very low endogenous expression level of APT-1 could be deacylating GAP-43, we evaluated the deacylation kinetics of ^N13^GAP-43(C3S) in CHO-K1 cells overexpressing or not human APT-1 fused to mCherry fluorescent protein (hereafter APT-1, Fig. 3C and D). The deacylation kinetics of GAP-43 was evaluated by analyzing the rate of ^N13^GAP-43(C3S)-TGN-membrane association decayment after 2-BP treatment. Results clearly indicated that APT-1 overexpression in CHO-K1 cells did not significantly modify the decay rate of ^N13^GAP-43(C3S) at the TGN compared to cells only expressing ^N13^GAP-43(C3S). Additionally, we also found that APT-1 overexpression did not significantly modify membrane association and deacylation kinetic of diacylated ^N13^GAP-43 and GAP-43^full^ (Fig. S3). To further analyze the effect of APT-1 activity on ^N13^GAP-43(C3S), we performed an in vitro deacylation assay (Fig. 3F). For these experiments, cytosolic fractions were prepared from CHO-K1 cells expressing ^N13^GAP-43(C3S) or a fusion protein containing the C-terminal hypervariable region of K-Ras fused to YFP (K-Ras^C14^), and particulate fractions from nontransfected cells [47]. Soluble fractions from transfected cells were incubated with membranes from nontransfected cells for 1 h at 4°C and then ultracentrifuged to separate soluble and particulate fractions. The isolated particulate fraction containing monoacylated GAP-43 or prenylated K-Ras was incubated for 1 h at 30°C in the presence (+APT-1) or the absence (-APT-1) of recombinant APT-1 [13]. Next, soluble and particulate fractions were separated by ultracentrifugation and presence of ^N13^GAP-43(C3S) in the fractions evaluated by Western blot. Results indicated that the incubation with recombinant APT-1 did not significantly increase the amount of ^N13^GAP-43(C3S) recovered in the soluble fraction. As expected, the amount of nonpalmitoylated K-Ras isoform present in the soluble fraction was independent of the presence of APT-1 (result not shown). Thus, these results strongly support the concept that APT-1 does not catalyze both in vitro and in vivo ^N13^GAP-43(C3S) deacylation.

As additional control of APT-1 activity, we evaluated the effect of ectopically expressed APT-1 on H-RasG12V deacylation and its consequences on subcellular distribution. APT-1 has been identified as one of the thioesterases in the acylation cycle of H-Ras both in vivo [21], [50] and in vitro [13]. In addition, it was demonstrated that oncogenic H-RasG12V is deacylated faster than its wild-type counterpart [15]. As shown in Figure 3E, H-RasG12V localizes at the plasma membrane and perinuclear structures previously identified as RE [42]. Treatment of CHO-K1 cells with 2-BP for 3 h led to a redistribution of H-RasG12V to perinuclear structures, which resembles the Golgi complex and the endoplasmic reticulum in agreement with subcellular distribution observed for nonpalmitoylated H-Ras [19], [22], [51]. A total redistribution of H-RasG12V to endomembranes was observed at 6 h after 2-BP treatment. By the contrary, when CHO-K1 cells coexpressing APT-1 and H-RasG12V were incubated with 2-BP, a faster and marked H-Ras subcellular redistribution was evident at 3 h. This result demonstrates that APT-1 is able to participate in the acylation cycle of H-RasG12V, which is consistent with already published data [50]. Altogether, these results indicate that APT-1 is not the enzyme responsible for GAP-43 deacylation in CHO-K1 cells. As CHO-K1 cells do not naturally express APT-1, the results strongly suggest the existence of other uncharacterized acyl-protein thioesterase.

### Bioinformatic Search and Expression Profiles Define Acyl-Protein Thioesterase 2 (APT-2) as the Enzyme Responsible for GAP-43 Deacylation in CHO-K1 Cells

**Figure 4:**
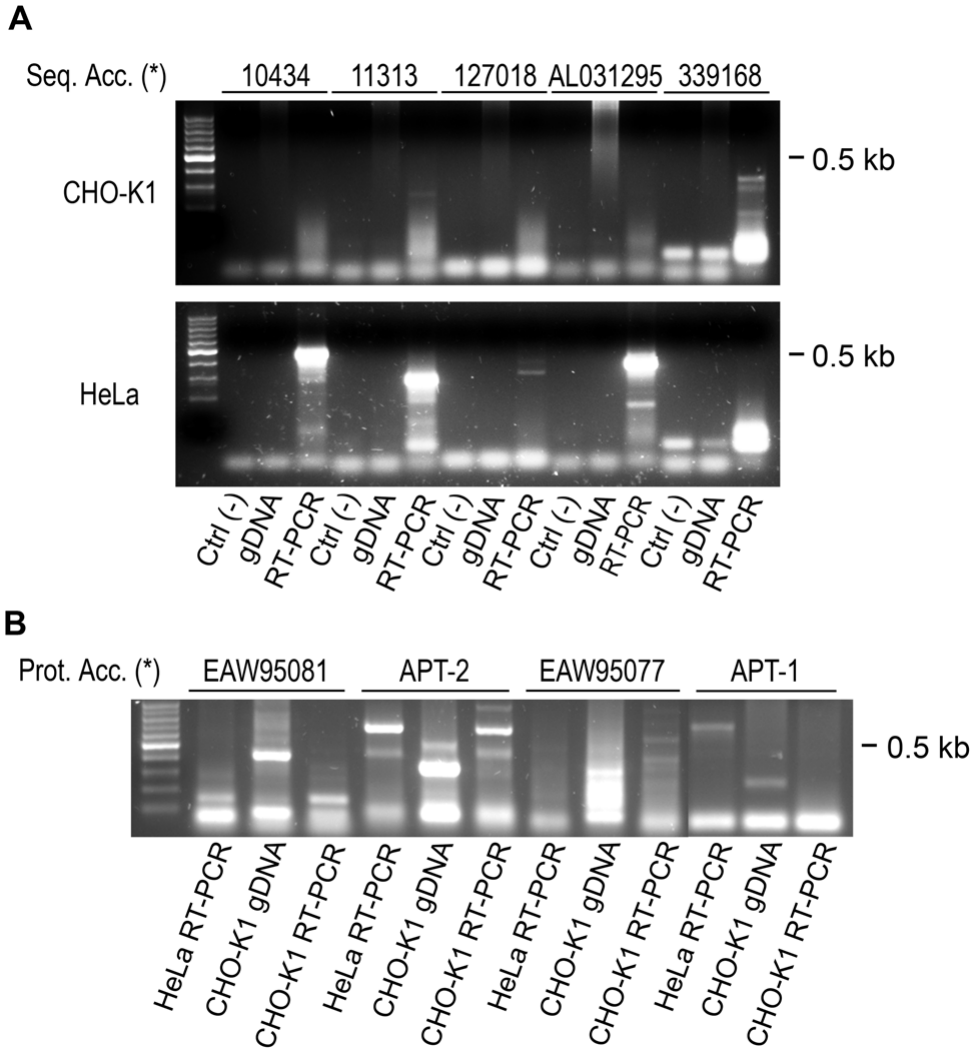
Analysis of acyl-protein thioesterase expression in CHO-K1 and HeLa cells. **A**) PCR analysis of gene expression in CHO-K1 (upper panels) and HeLa cells (lower panels). For each indicated gene (see gene number at the top), PCRs were performed using specific primers and the following templates: reaction mixture [Ctrl (-)]; genomic DNA (gDNA) and first-strand cDNA obtained from RT of mRNA (RT-PCR). **B**) PCR analysis of isoform expression coded by gene # 11313. For each isoform indicated at the top, PCRs were performed using specific primers and the following templates: first-strand cDNA obtained from RT of HeLa cells purified mRNA (HeLa RT-PCR); CHO-K1 genomic DNA (CHO-K1 gDNA) and first-strand cDNA obtained from RT of CHO-K1 cells purified mRNA (CHO-K1 RT-PCR).

To identify the enzyme/s responsible for GAP-43 deacylation in CHO-K1 cells, we performed a bioinformatic search based on shared properties of the only two proteins described with APT activity, APT-1 and PPT-1. First, we performed a Blast search for nonredundant human protein sequences which align to PPT-1 and/or APT-1 protein sequences. In addition, we searched for members of APT-1 and/or PPT-1 families in Pfam databases (http://pfam.sanger.ac.uk/) [#PF02230 (APT-1) and #PF02089 (PPT-1)]. After careful classification and removal of redundant sequences, the in silico analysis allowed us to identify 21 potential acyl thioesterase transcripts, which are codified for 5 different genes (Table S1). To simplify the experimental screening, we first determined transcript expression in CHO-K1 and HeLa cells by RT-PCR using specific primers for each gene (without discrimination of mRNA isoforms, Fig. 4A). In parallel, we also determined transcription profiles in the neuroblastoma cell line SH-SY5Y (undifferentiated and retinoic acid-differentiated) and SK-Mel28 cells (Fig. S4).

We found that transcripts coded by gene ID 11313 were expressed in much higher amount in HeLa, SH-SY5Y and SK-Mel-28 cells than in CHO-K1 cells. Interestingly, we observed that the expression level of transcripts coded for this gene increased in retinoic acid-treated SYSH-5Y cells when compared to control conditions. In addition, transcripts coded by gene ID 127018 and gene ID 339168 were only found in neuronal (SH-SY5Y) and epithelial (CHO-K1) cells, respectively. Finally, transcripts coded by gene ID 10434 were found in all analyzed cell lines, except CHO-K1 cells. Thus, the transcription profile of genes ID 127018, 339168 and 10434 did not explain for the presence of acyl-protein thioesterase activity both in CHO-K1 and HeLa cells. Consequently, results strongly suggest that gene ID 11313 potentially code for an acyl-protein thioesterase for GAP-43.

To identify transcripts derived from gene ID 11313 potentially coding for GAP-43 acyl-protein thioesterases, we evaluated their expression both in CHO-K1 and HeLa cells by RT-PCR using specific primers for each transcript (Fig. 4B). Results show that APT-2 is the only isoform expressed both in CHO-K1 and HeLa cells, and suggest that it could be a potential candidate involved in GAP-43 deacylation. Sequence analysis revealed a high degree of conservation between APT-2 from CHO-K1 and HeLa cells (aminoacid identity: 99%; nucleotide sequence 93%) (Table S2).

### APT-2 Overexpression Increases the Deacylation Rate of GAP-43

**Figure 5:**
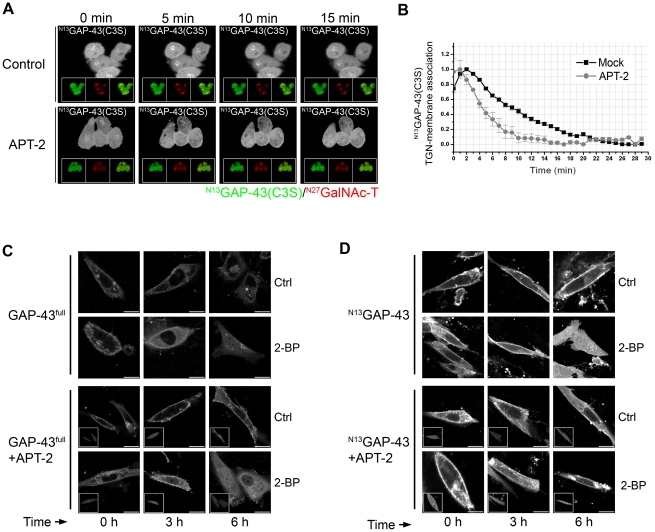
APT-2 overexpression increases the deacylation rate of GAP-43. **A**) 60 h after transient transfection, CHO-K1 cells expressing ^N13^GAP-43(C3S) (Control, upper panels) or coexpressing ^N13^GAP-43(C3S) and APT-2 (APT-2, lower panels) were treated with 50 µM 2-BP. CHX and protein degradation inhibitors were added 30 min before imaging and maintained in the culture media until the end of each experiment. GAP-43 subcellular distribution was analyzed by live cell confocal microscopy. Each panel shows image from YPF fluorescence (pseudocolored gray) at 0, 5, 10 and 15 min from 2-BP addition. The insets in each panel from left to right show: YFP fluorescence (pseudocolored green), CFP fluorescence (pseudocolored red) and the merge of both fluorescence signals. **B**) Quantification [^N13^GAP-43(C3S) associated to TGN membranes] of images showed in panels a (see Materials and Methods). **C**) 60 h after transient transfection, CHO-K1 cells expressing GAP-43^full^ (upper panels) or coexpressing GAP-43^full^ and APT-2 (lower panels) were treated with 50 µM 2-BP (2-BP) or vehicle (Ctrl) in the presence of CHX and protein degradation inhibitors for 0, 3 and 6 h and the GAP-43 subcellular distribution was analyzed by live cell confocal microscopy. Each panel shows image from YFP fluorescence (pseudocolored gray). The insets show cells expressing APT-2 (cherry fluorescence, pseudocolored gray). **D**) 60 h after transient transfection, CHO-K1 cells expressing ^N13^GAP-43 (upper panels) or coexpressing ^N13^GAP-43 and APT-2 (lower panels) were treated with 50 µM 2-BP (2-BP) or vehicle (Ctrl) in the presence of CHX and protein degradation inhibitors for 0, 3 and 6 h and the subcellular distribution was analyzed by live cell confocal microscopy. Each panel shows image from YFP fluorescence (pseudocolored gray). The insets indicate cells expressing APT-2 (cherry fluorescence, pseudocolored gray). Scale bars: 5 µm.

Bioinformatic search and mRNA expression experiments suggested that APT-2 is a potential acyl-protein thioesterase for GAP-43. To test this hypothesis, we evaluated the effect of APT-2, fused to mCherry fluorescent protein (hereafter APT-2), overexpression on ^N13^GAP-43(C3S) deacylation in CHO-K1 cells by live cell confocal microscopy as described in Fig. 2. As control, we also measured ^N13^GAP-43(C3S) deacylation in cells which did not overexpress APT-2 (Fig. 5A). As previously determined, the half-time of the decay of TGN-membrane associated GAP-43 at the TGN after 2-BP addition in cells which only expresses ^N13^GAP-43(C3S) was 6 min (see Fig. 2). In a clear contrast, we observed that in cells expressing APT-2, the half-time of ^N13^GAP-43(C3S) deacylation was reduced to 3 min (Fig. 5B), demonstrating that APT-2 catalyze in vivo deacylation of ^N13^GAP-43(C3S).

To further extent our research, we investigated the effect of APT-2 overexpression on GAP-43^full^ and ^N13^GAP-43 deacylation. For this, we analyzed the effect of 2-BP treatment on GAP-43 subcellular distribution in cells expressing GAP-43 alone or coexpresing GAP-43 and APT-2 (Fig. 5C and D). We observed that in the absence of APT-2, GAP-43 (^N13^GAP-43 and GAP-43^full^) deacylation was partially evident after 3 h of 2-BP treatment (compare Control with 2-BP condition), maintaining a significant amount of plasma membrane-associated GAP-43. In contrast, after 6 h of 2-BP treatment, we observed nuclear accumulation of soluble GAP-43 and a clear reduction of GAP-43 at the plasma membrane, which indicate that it is highly deacylated under this condition. On the other hand, the overexpression of APT-2 caused a significant reduction in the plasma membrane content of GAP-43^full^ at 3 h of 2-BP treatment (Fig. 5C). In addition, it was also observed at this time an appreciable accumulation of soluble GAP-43 in the nuclei when compared to cells expressing only GAP-43. A similar result was also observed when we analyzed the effect of APT-2 overexpression on ^N13^GAP-43 deacylation (Fig. 5D). Altogether, these results indicate that APT-2 is a novel acyl-protein thioesterase which is able to deacylate GAP-43.

### Effect of APT-2 Overexpression on the Deacylation Rate of H-RasG12V

**Figure 6:**
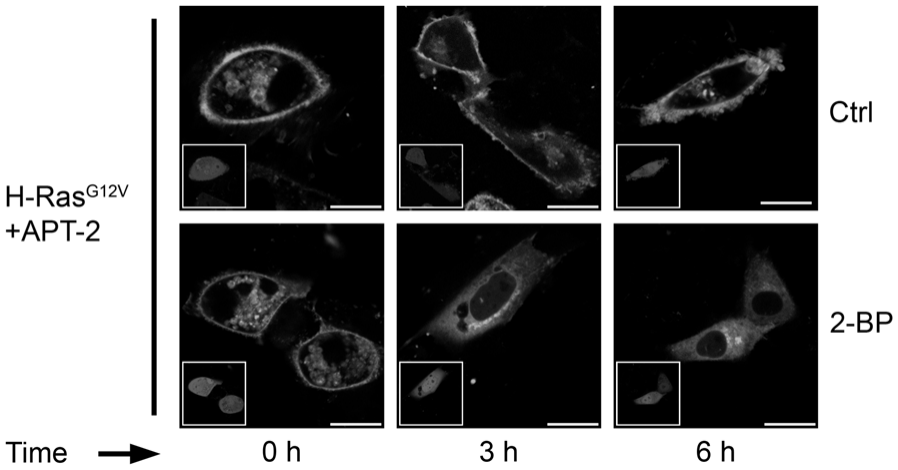
Effect of APT-2 overexpression on the deacylation rate of H-RasG12V. 60 h after transient transfection, CHO-K1 cells coexpressing H-RasG12V and APT-2 were treated with 50 µM 2-BP (2-BP) or vehicle (Ctrl) in the presence of CHX and protein degradation inhibitors for 0, 3 and 6 h and H-RasG12V subcellular distribution analyzed by live cell confocal microscopy. Each panel shows image from YFP fluorescence (pseudocolored gray). The insets show cells expressing APT-1 (cherry fluorescence). Scale bar: 5 µm.

After identifying GAP-43 as a substrate of APT-2, we investigated the effect of APT-2 expression on H-RasG12V deacylation and subcellular distribution (Fig. 6). Similar to results obtained in CHO-K1 cells coexpressing APT-1 and H-RasG12V (Fig. 3E), APT-2 expression caused a total H-RasG12V redistribution to the endoplasmic reticulum after 3 h of 2-BP treatment. Thus, results demonstrate that H-RasG12V deacylation rate in cells coexpressing APT-2 is much higher than the observed in cells expressing H-RasG12V alone (see Fig. 3E) and suggest that, like APT-1, APT-2 is also able to deacylate H-Ras in vivo.

## Discussion

The major aim of this study was to gain insight into the mechanisms participating in GAP-43 deacylation. Using confocal and video fluorescence microscopy in living cells, in association with biochemical approaches, we demonstrated in HeLa and CHO-K1 cells that both ^N13^GAP-43 and GAP-43^full^ are completely deacylated in vivo in a scale time of hours. In addition, single acylated mutants of GAP-43 were found to be deacylated faster than their double acylated counterparts. GAP-43 deacylation was not catalyzed by APT-1, the only bona fide cytosolic protein thioesterase described so far. By bioinformatic search, molecular biology techniques and later cell imaging analysis, we identified and characterized the phospholipase like protein APT-2 as the enzyme involved in GAP-43 deacylation.

It had been early demonstrated that GAP-43 is deacylated in vivo [21], [22], [40], [41] and that at steady-state conditions there are a variety of fatty acylated species of GAP-43, including, non-, single and double acylated forms [46]. Although in vitro experiments shown that an acylated peptide substrate derived from GAP-43 (MLCCMRR) was efficiently deacylated by PPT-1 at neutral pH [52], the lysosomal localization of this enzyme preclude the possibility that it is involved in cytosolic GAP-43 deacylation [21], [53]-[55]. In opposite, APT-1, originally isolated from rat liver as a lysophospholipase [27], appears to be involved in the regulation of the reversible S-acylation of cytoplasmic proteins in vivo, like heterotrimeric G protein α subunits, endothelial nitric-oxide synthase, SNAP-23 and H-Ras as well as viral proteins [18]. However, evidence also indicates that APT-1 does not deacylate proteins without any discrimination. For instance, caveolin is not deacylated by APT-1 [56] and not all substrates are deacylated with the same efficiency [57]. Regarding GAP-43 deacylation, there is not any direct evidence suggesting the participation of APT-1.

Taken into consideration these antecedents, we attempted to evaluate the role of APT-1 in GAP-43 deacylation. First of all, we demonstrated that wild-type or single acylation mutants of GAP-43 are deacylated in CHO-K1 cells. This result is compatible with those recently published [21] and in agreement with previous reports [40], [41]. Interestingly, we demonstrated that APT-1 is not expressed in CHO-K1 cells and that APT-1 overexpression did not significantly increase the deacylation rate of monoacylated or wild-type GAP-43 but did enhance the deacylation rate of H-RasG12V protein. Paradoxically, APT-1 overexpression caused an accumulation of GAP-43^full^ at the Golgi complex, a phenomena which could be compatible with an increase in acylation activity probably mediated by APT-1 since under the experimental conditions used in Figure S3 endogenous PAT activity, but not APT-1, was inhibited at 50 µM 2-BP (Tomatis, Gomez and Daniotti, unpublished results). In this sense, it was previously described that APT-1 may not only function as a thioesterase but also as an acyltransferase. Particularly, it was demonstrated an APT-1-mediated S-palmitoylation of H- and N-Ras [45]. Additionally, the no accumulation of ^N13^GAP-43 at the Golgi complex in APT-1 expressing CHO-K1 cells could be reflecting substrate selectivity or structural preference of APT-1 to exert a proper acylation/reverse hydrolysis.

To identify the enzyme/s responsible for GAP-43 deacylation, we performed a bioinformatic search based on shared properties of the only two proteins described with APT activity, APT-1 and PPT-1. Results show that APT-2 is the only isoform expressed both in CHO-K1 and HeLa cells, and suggested that it could be a potential candidate involved in GAP-43 deacylation. Subsequent experiments using live cell imaging analysis confirmed the bioinformatic results and demonstrated for the first time that APT-2 behave as acyl-protein thioesterase which is able to deacylate either GAP-43^full^ or ^N13^GAP-43.

Mouse APT-2, which shares 64% identity with rat and mouse APT-1, was found to be ubiquitously expressed in mouse tissues and able to hydrolyze several lipid substrates with varied efficiency [58], [59]. Sequence analysis using the PSORTII program (http://psort.hgc.jp/form2.html) indicates that APT-2 is likely a soluble protein. By fusing the cDNA encoding this protein to fluorescent proteins, we found that APT-2 is mostly cytosolic and there is not any apparent membrane association. This fact strongly suggests that APT-2 can deacylate GAP-43 by transient binding to the membrane-cytosol interface probably in a widely distributed process. In particular, we observed that deacylation of monoacylated ^N13^GAP-43 occurs as early in the acylation cycle as at the Golgi complex. In agreement with this result, it was recently demonstrated that palmitate removal of H-Ras mutants (mono and double-palmitoylated) takes place at the Golgi complex [21].

Although some patterns of S-acylation exist, there is no specific consensus sequence for S-acylation. Similarly, it has not been defined so far a consensus for the sequences surrounding the thioacyl group which could be recognized by APT-1. In vitro, APT-1 can depalmitoylate structurally different proteins (soluble or integral membrane proteins), having different subcellular distributions and S-acylated either at the N- (i.e. endothelial nitric-oxide synthase and G_iα1_) or at the C-terminus (i.e. H-Ras) [18]. We observed that, like APT-1, APT-2 has not an easily detected consensus sequence for deacylation. We demonstrated that APT-2 can deacylate two unrelated proteins like H-Ras, which is modified at its C-terminus by both acylation and prenylation and GAP-43, which is acylated on N-terminal cysteines 3 and 4. Moreover, we observed variable substrate preference for APT-2 when compared deacylation of GAP-43^full^ and the acylation motif of GAP-43 fused to YFP.

Concerning APT-2 deacylation of H-Ras, it was early speculated it could be occurring in yeast cells where the APT-1 gene was disrupted since the in vitro acyl-protein thioesterase activity against acylated H-Ras was similar to that of wild-type yeast [57]. In this sense, our results agree with this hypothesis and support the fact that both APT-1 and APT-2 mediate H-Ras deacylation. However, bioinformatic search do not indicate that APT-2 homologue exists in yeast, suggesting that in this organism Ras deacylation might be catalyzed by another still unidentified thioesterase protein [18].

Previously, it was demonstrated that proteasome inhibitors increase the cellular GAP-43 level, leading to the accumulation of polyubiquitinated forms of this protein in transfected cells. Moreover, the ubiquitin-proteasome pathway was described to be important in the turnover of this protein in neuron cells [43], [44]. In this work, we show that 2-BP treatment caused a significative reduction in the amount of transiently expressed GAP-43. Interestingly, we demonstrated that the reduction in the protein level was abrogated when cells were also treated with proteasome inhibitors (chloroquine, MG132 and lactacystin) which strongly suggest that GAP-43 deacylation is an early and necessary step for its later ubiquitination and degradation by the proteasome. In addition, it also suggests that acyl-protein thioesterase levels not only regulate palmitate turnover but also global protein turnover of GAP-43.

## Materials and Methods

### Plasmids

The expression vectors pECFP-C1 (where ECFP, enhanced CFP), pEYFP-N1 (where EYFP, enhanced YFP), pECFP-C1, pEYFP-C1, pEGFP-F, pmCherry-C1 and the plasmid encoding GFP-H-Ras^C20^ were from Clontech (CA, USA). Expression plasmids for ^N13^GAP-43-(YFP), GAP-43^full^-(YFP) and its acylation mutants, (YFP)-K-Ras^C14^, YFP-H-Ras^full^, ^N27^GalNAc-T-CFP and ^N52^Gal-T2-CFP have been previously described [34], [42], [47], [60]. The expression plasmid encoding YFP-H-Ras^G12V^ was performed by directed mutagenesis of YFP-H-Ras^full^ using PCR. Both Cherry-APT-1 and Cherry-APT-2 constructs were obtained by RT-PCR using purified mRNA from HeLa and CHO-K1 cells, respectively, and the amplified products were cloned into pmCherry-C1 using EcoRI and SalI restriction sites.

### Cell Culture and DNA Transfections

CHO-K1, HeLa, SK-Mel 28 and SHSY-5Y cells (ATCC, Manassas, VA, USA) were maintained at 37°C, 5% CO_2_ in Dulbecco's modified Eagle's medium (DMEM) supplemented with 10% fetal bovine serum (FBS) and antibiotics (ATB, 100 µg/ml penicillin and 100 µg/ml streptomycin). To induce SH-SY5Y differentiation, cells were incubated with 30 µM retinoic acid for 6 days. Cells grown on Petri dishes were used for both live cell imaging and Western blot experiments. Cells were transfected with 0.6–1.2 µg/35 mm dish of the indicated plasmid using cationic liposomes (Lipofectamine, Invitrogen, CA, USA) or polyethyleimine (PEI, Sigma-Aldrich, St Louis, MO, USA). At 24 h after cell transfection, cells were processed for Western blot experiments or plated onto coverslips, incubated for 24 h, and used in live cell imaging.

### 2-BP Treatment

The stock solution of 2-BP (0.42 M, Fluka) was prepared in DMSO. CHO-K1 and HeLa cells expressing recombinant proteins were incubated with 50 µM 2-BP or vehicle (DMSO) for control cells for the indicated times. To analyze the effect of 2-BP on the steady state subcellular distribution of fluorescent proteins, CHO-K1 and HeLa cells were treated with 60 or 150 µg/ml CHX (Sigma-Aldrich), respectively, and inhibitors of protein degradation (60 µM cloroquine and 7.5 µM MG132). These inhibitors were added to the culture medium 24 h after cell transfection and 1 h before 2-BP treatment (Western blot experiments) or 36 h after platting transfected cells onto coverslips (live cell imaging). In live cell imaging experiments, coverslips were mounted on the microscope stage and 2-BP was added during time series acquisition.

To analyze the effect of acylation inhibition on membrane binding properties of GAP-43, 50 µg/ml CHX was incorporated to the culture medium during the transfection. Then, 5 h after transfection, 10% FBS was added to the medium and 2h later 50 µM 2-BP was also incorporated. After 1 h of 2-BP treatment, CHX was removed by three washes with DMEM containing FBS, antibiotics and 2-BP. Finally, after 4 h of CHX withdrawal, cells were scrapped and used for biochemical experiments.

### Electrophoresis and Western Blotting

Electrophoresis, transfer onto nitrocellulose membranes and protein immunodetection were performed as described by [47]. Anti-GFP polyclonal antibody (Roche Diagnostics, IN, USA) was used at a dilution of 1∶700. Antibodies were detected using near-infrared fluorescence (Li-COR Biotechnology, Lincoln, NE, USA) with secondary antibody coupled to IRDye800CW (Li-COR Biotechnology). The relative contribution of individual bands was calculated using the computer software ImageJ (National Institute for Health, Bethesda, MA, USA) on raw nonsaturated digital images and the ^N13^GAP-43 *Kp* value determined as follow:
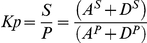



Where S and P refer to the amount of GAP-43 present in S [Aqueous (A) plus Detergent (D) fractions] and P (A plus D fraction), respectively, after background subtraction. Values of *Kp* were normalized to those observed in control conditions.

### Subcellular Fractionation and TX-114 Partition Assay

Cells grown onto 60 mm dishes were washed with cold PBS (140 mM NaCl, 8.4 mM Na_2_HPO_4_, 1.6 mM NaH_2_PO_4_, pH 7.5); and harvested by scrapping in PBS containing a protease inhibitor mixture (PIM) containing 5 µg/ml aprotinin, 0.5 µg/ml leupeptin, and 0.7 µg/mL pepstatin (PBS-PIM). Extracts were centrifuged at 4°C for 5 min at 13000 *g* and resuspended in 400 µl of 5 mM Tris-HCl (pH 7.0) (buffer T) in the presence of protease inhibitors (T-PIM). Pellets were dispersed by repeated pipetting and vortexing. After 30 min of incubation in T-PIM, pellets were passed 60 times through a 25-gauge needle. Nuclear fractions and unbroken cells were removed by twice centrifugation at 4°C for 5 min at 600 *g*. Supernatants were then ultracentrifuged at 4°C for 1 h at 400000 *g* using a TLA 100.3 rotor (Beckman Coulter, Inc., CA, USA). The supernatant (S1 fraction) was removed, and the pellet (P1 fraction) was resuspended in 400 µl of T-PIM. Both fractions were further ultracentrifuged at 400000 *g* and the supernatant of S1 fraction was removed (S fraction) and the pellet of P1 fraction was resuspended in 400 µl of T-PIM (P fraction). 100 µl of 5% (v/v) TX-114 in T-PIM was added to S and P fractions and incubated at 4°C during 1 h. Then, samples were incubated at 37°C for 3 min and centrifuged at 13000 *g*. The aqueous upper phase (A) and the detergent-enriched lower phase (D) were separated and extracted again with detergent and aqueous solutions, respectively. The resulting samples were adjusted to equal volumes and detergent content, and proteins were precipitated with chloroform/methanol (1∶4 v/v) for Western blot analyses.

### Expression and Purification of Recombinant APT-1

The APT-1 cDNA containing a Hisx6 tag was obtained by RT-PCR using specific primers and RNA from HeLa cells. The amplified fragment was cloned into BamHI/EcoRI sites of the bacterial expression vector pRSET-A (Invitrogen, USA). Transformed *Escherichia coli* cells were growth at 37°C in LB medium containing 75 µg/ml ampicilin to an optical density of 0.6. Then, a soluble fraction was generated by sonication, lyzosyme treatment (200 µg/ml, 30 min at room temperature), and centrifugation at 10000 g for 15 min at 4°C. Hisx6-APT-1 was then purified from the soluble lysate using a Ni^2+^-NTA column (GE Healthcare, Fairfield, VT, USA) according to manufacturer's instructions.

### Assessment of ^N13^GAP-43(C3S) In Vitro Depalmitoylation by Purified APT-1

To examine the ability of purified recombinant APT-1 to depalmitoylate ^N13^GAP-43(C3S), S and P fractions were obtained from ^N13^GAP-43(C3S) transfected and nontransfected CHO-K1 cells, respectively, by ultracentrifugation as described above. S_0_ fractions were cleared and P_0_ fractions were washed by ultracentrifugation. Membrane binding of soluble ^N13^GAP-43(C3S) protein was done by incubation of S_0_ fractions obtained from transfected cells with P_0_ fractions obtained from untransfected cells for 1 h at 4°C. The resultant mixture was then ultracentrifuged for 1 h at 4°C and the pellet fraction (P_1_) was resuspended in 400 µl APT-1 reaction buffer (50 mM Hepes, 2 mM CaCl_2_, 0.1 mM EDTA, pH 8.0) containing protease inhibitors (PIM plus 1 mM PMSF) and ultracentrifuged again. The resultant P_2_ fraction was resuspended in 250 µl APT-1 reaction buffer containing PIM/PMSF and subjected to acyl-protein thioesterase treatment by incubation with recombinant APT-1 as previously described [13], [14], [25] with some modifications. Reactions were performed by mixing 100 µl of P_2_ fraction with 2 µg recombinant APT-1 (0.18 µg/µl; APT-1 reactions) or 11.1 µl of APT-1 carrier buffer (1M Tris-HCl:glycerol, 1∶1; Control reactions) and incubated at 30°C for 1 h. Then, 300 µl of APT-1 reaction buffer was added to samples and subjected to ultracentrifugation. P fractions were then resuspended in the same reaction buffer. Proteins from resulting fractions were precipitated with 10% trichloroacetic acid as described previously [61] and analyzed by Western blot using an antibody to GFP. As a control, we also evaluated under the same experimental conditions the effect of recombinant APT-1 on membrane binding properties of YFP-K-Ras^C14^ [47].

### Confocal Microscopy

Confocal images were collected using an Olympus FluoView FV1000 confocal microscope (Olympus Latin America, Miami, FL) equipped with a multi-line Argon laser (458, 488 and 514 nm), two Helium Neon lasers (543 nm and 633 nm, respectively). CFP was detected by using laser excitation at 458 nm; a 458/514 nm excitation dichroic mirror and a 470–500 nm bandpass emission filter. YFP was acquired by using laser excitation at 514 nm, a 458/514 nm excitation dichroic mirror and a 530–570 nm bandpass emission filter. Cherry protein was acquired with a laser excitation at 543 nm, a 488/543/633 nm excitation dichroic mirror, and a 560 nm long pass emission filter. For CFP/YFP or CFP/YFP/Cherry acquisition, images were sequentially acquired in line mode. This minimizes the bleed-through between channels, mainly due to overlapping emission spectra of these fluorochromes.

#### i) Live Cell Imaging for Subcellular Distribution Analyses

For subcellular distribution analyses and colocalization of GAP-43 proteins with organelle markers, live cell experiments were performed at 35±2°C (Tritech DigiTherm temperature controller and a heating adapter plate, CA, USA) and using a 100× UplanSApo oil immersion/1.4 NA (numerical aperture) objective (Olympus, Japan). Images were taken using 3 x digital zoom, and an appropriated pinhole to obtain 1 Airy unit for the fluorochrome of shortest wavelength excitation/emission properties (optical slice 0.8 µm). Images of different cells for each dish were taken in a period no longer than 30 min. Images are representative of at least three independent experiments.

#### ii) Live Cell Imaging for Deacylation Kinetic Measurements

For deacylation measurements, cells coexpressing ^N13^GAP-43(C3S)-YFP and ^N27^GalNAcT-CFP, a TGN marker, or ^N13^GAP-43(C3S)-YFP, ^N27^GalNAcT-CFP and Cherry-APT-1 or Cherry-APT-2, were used. Live cell experiments were performed at 20°C on the Olympus FluoView FV1000 confocal microscope to minimize vesicular trafficking of GAP-43 [34], [62]. After selection of cells coexpressing all proteins (e.g., ^N27^GalNAc-T-CFP, ^N13^GAP-43(C3S) and cherry-APT-1 or cherry-APT-2), images were acquired in the CFP and YFP channels for deacilation kinetic measurements. A 40 x/1.3 NA PlanApo objective oil immersion (Olympus) was used with a 5 x digital zoom. The image resolution was 320x320 pixels, the scan speed was 10 µs/pixel and the pinhole adjusted to obtain an optical slice of 3 µm. Single 2D images were taken at a frequency of 1 min^-1^, for 30 minutes. These acquisition conditions were optimized to acquire the Golgi compartment, to minimize bleaching during acquisition and to sample as fast as possible the amount of ^N13^GAP-43(C3S) at the TGN.

### Quantitative mage Analysis for Deacylation Experiments

All image analysis and quantification procedures were done using ImageJ software. Significant fluorescent pixels were isolated from raw images of each channel (CFP and YFP channels) by applying the following equation:
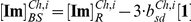



Where 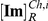 is the matrix (image) containing the gray values of the raw image of channel i, 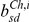 is a scalar representing the standard deviation of intensity of background pixels and [**1**] is a unitary matrix of equal size of 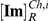. b_sd_ was obtained from a 50×50 pixel region from an area empty of cells. Negative elements resulting from the above equation are defined as 0 and consequently the elements of the 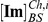 matrix are greater or equal to 0. The threshold 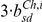
allows discriminate those pixels whose intensity is significantly different from the background noise. Although the intensity distribution of pixels is not normal, we did not obtain differences by applying the above equation or a threshold containing the 90% of background pixels (data not shown).

A mask 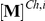, containing the position of significant pixels in 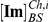 was generated by applying a threshold value equal to 1, and then substrating a value of 254 in Image J. The resulting image was then converted to 16 bit for compatibility purposes. The 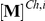 matrix has values 0 and 1. Then, objects with a diameter smaller than 8 pixels were discarded with the remove objects plugin of Image J. Once obtained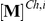, pixels_ij_ in the raw image with significant intensity values were selected by 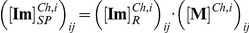



i.e, by multiplying raw and mask images using the image calculator tool.

For TGN masks (i.e. those derived from ^N27^GalNAcT-CFP images) the background level in the CFP channel was determined around the organelle and not from an area empty of cells since in our conditions a fraction of ^N27^GalNAc-T-CFP was present in the endoplasmic reticulum. After creating each TGN mask, we measured the area of TGN in pixel number units as:
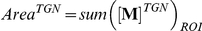



Where TGN refers to the CFP channel. The sum of the elements of the mask was performed over a ROI that completely involves the organelle in all the time series of a single cell.

By multiplying each ij pixel of the 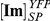
(the 2-D matrix containing the significant ^N13^GAP-43(C3S)-YFP data) by the corresponding of 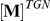, we obtain
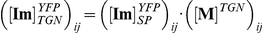



and the integrated YFP fluorescence intensity inside the organelle is obtained by
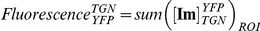



Where the ROI is the same that was employed to determine TGN area. The ^N13^GAP-43(C3S)-YFP concentration is determined by dividing 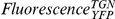 by 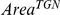.
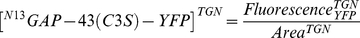



Because we were interested how GAP-43 is distributed between TGN and cytoplasm and the above parameters scaling up with protein expression, we measured the TGN to cytoplasm concentration ratio at each time *i* as follow:
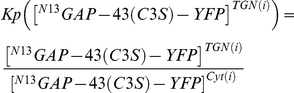



Where TGN and Cyt refers to the TGN and cytoplasm, respectively. The concentration of GAP-43 at the cytoplasm was determined by measuring the average gray value (integrated gray value/number of pixels of the ROI) of GAP-43 in a small region of interest located in the cytoplasm. Typically, the cytoplasmic ROI area was comparable to that of TGN marker area. Thus, data acquisitions are independent of organelle size and initial steady state conditions. Moreover, these parameters were also independent of GAP-43 expression levels and TGN size, as previously described [34].

For GAP-43 deacylation, we considered that after an infinite period of acylation inhibition in the presence of an active deacylation mechanism, the amount of TGN associated GAP-43 completely vanish. At this point, GAP-43 is completely dissociated from TGN and only localizes at the cytosol as a soluble protein. For this reason, we expect that the *Kp* parameter reach 1 at this time. In this sense, it was considered more appropriate to describe the acylation state measured as the amount of TGN-membrane associated GAP-43 as follow:
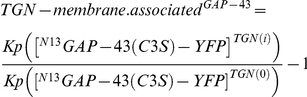



Where 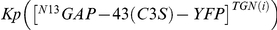 and 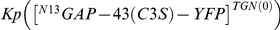 are the TGN to cytoplasm concentration ratios of ^N13^GAP-43(C3S) at time i and 0, respectively.

### mRNA Purification and RT-PCR

Animal handling was performed according to the standards stated in the Guide to the Care and Use of Experimental Animals published by the Canadian Council on Animal Care and approved by the local Institution (Facultad de Ciencias Químicas, Universidad Nacional de Córdoba, Argentina, Exp. N° 15-99-40426).

For hamster tissue mRNA purification and analyses, hamsters were anesthetized and different organs extracted. Then, the tissues were homogenized with an Ultraturrax tissue disintegrator and lysates were passed through a Dounce homogenizer until homogeneity. Then, the cleared lysates were centrifuged at 13000 g and mRNA or genomic DNA were extracted using mRNA and genomic DNA extraction kits from GE healthcare and Sigma-Aldrich, respectively.

For tissue cultured cells, 24 h after seeding or differentiation, cells were washed with cold phosphate buffered saline (PBS) and harvested by scrapping in PBS. Extracts were centrifugated at 13000 g for 15 min and mRNA or genomic DNA purified using the commercial extraction kits, according to manufacture instructions. After purification, reverse transcriptase-PCR was performed using 2 µg of purified mRNA as template. Alternatively, PCR was performed using 50 ng of genomic DNA. Primers are described in supplemental Table S1.

### Bioinformatic Search for Acyl-Protein Thiosterases

First, we performed a Blast search for nonredundant human protein sequences (nonredundant human database) which align to PPT-1 (NP_000301.1) and/or APT-1 (NP_006321.1) protein sequences [63], [64]. In addition, we attempted to identify members of PPT-1 and/or APT-1 families in Pfam databases (http://pfam.sanger.ac.uk/) [#PF02230 (APT-1) and #PF02089 (PPT-1)] [65]. Overall, we obtained four groups of proteins. After discarding redundant sequences, the resulting proteins were grouped according to their coding genes. Finally, we obtained five different genes coding for 21 mRNA transcripts which are potentially involved in the expression of acyl-protein thioesterase enzymes (Table S1).

## Supporting Information

Figure S1 **In vivo deacylation of single acylated GAP-43^full^(C3S).** **A**) CHO-K1 cells transiently expressing GAP-43^full^(C3S)-YFP [GAP-43^full^(C3S)] were treated with 50 µM 2-BP (2-BP) or vehicle (Control) for 2 h. Then, cells were lysed, ultracentrifuged and the S (soluble) and P (pellet) fractions isolated. Buffer containing 1% v/v TX-114 was added to samples and the phase separation was induced at 37°C. Proteins from the A (aqueous) and D (detergent) phases were Western blotted with an antibody to GFP. The lowest panels show the Western blot using antibody to α-tubulin. **B**) Quantification of Western blot showed in A (see Materials and Methods). Kp = K1/K2, where K1 = P GAP-43/S GAP-43 and K2 = P tubulin/S tubulin. Kp control = 100%.
(TIF)

Figure S2 **APT-1 expression analysis in COS-7 and CHO-K1 cells.** PCR analysis of APT-1 expression using the following templates: first-strand cDNA obtained from RT of CHO-K1 cells purified mRNA (RT-PCR CHO-K1); the reaction mixture [Ctrl (-)]; total mRNA from CHO-K1 cells (Total RNA CHO-K1); first-strand cDNA obtained from RT of COS-7 cells purified mRNA (RT-PCR COS-7); genomic DNA from CHO-K1 cells (gDNA CHO-K1) and from COS-7 cells (gDNA COS-7). Genomic DNA and mRNA were extracted from CHO-K1 and COS-7 cells 2 day after seeding in 100 mm Petri dishes.
(TIF)

Figure S3 **APT-1 overexpression did not significantly modify membrane association and deacylation kinetic of diacylated ^N13^GAP-43 and GAP-43^full^.** 60 h after transient transfection, CHO-K1 cells coexpressing GAP-43^full^ and APT-1 (**A**) or ^N13^GAP-43 and APT-1 (**B**) were treated with 50 µM 2-BP (2-BP) or vehicle (Ctrl) in the presence of CHX and protein degradation inhibitors for 0, 3 and 6 h and the GAP-43 subcellular distribution was analyzed by live cell confocal microscopy. Each panel shows image from YFP fluorescence (pseudocolored gray). The insets show the cells expressing APT-1 (cherry fluorescence, pseudocolored gray). Scale bar: 5 µm.
(TIF)

Figure S4 **Analysis of acyl-protein thioesterase expression in SH-SY5Y and SK-Mel 28 cells.** **A**) PCR screening of acyl-protein thioesterase gene expression in SH-SY5Y, treated (RA) or not (Ctrl) with retinoic acid (middle and upper panels, respectively), and SK-Mel 28 human cells (lower panels). For each indicated gene (see gene accession number at the bottom), PCRs were performed using specific primers and the following templates: reaction mixture [Ctrl (-)]; SH-SY5Y or SK-Mel 28 genomic DNA (gDNA) and first-strand cDNA obtained from RT of SH-SY5Y or SK-Mel 28 cells purified mRNA (RT-PCR). **B**) PCR analysis of isoform expression coded by gene # 11313. For each isoform indicated at the right (with the corresponding accession number), PCRs were performed using specific primers and the following templates: first-strand cDNA obtained from RT of SH-SY5Y [treated (RA) or not (Ctrl) with retinoic acid] or SK-Mel 28 cells purified mRNA (RT-PCR); SH-SY5Y or SK-Mel 28 genomic DNA (gDNA).
(TIF)

Table S1 **Bioinformatic data of genes potentially coding acyl-protein thioesterases and PCR primer sequences.**
(DOC)

Table S2 **Aminoacid and nucleotide sequence alignments of APT-2 from CHO-K1 and Hela cells.**
(DOC)
