# Supplementary figures and images for: Acyl-Protein Thioesterase 2 Catalizes the Deacylation of Peripheral Membrane-Associated GAP-43
Source: PLoS One. 2010 Nov 30;5(11):e15045. doi: 10.1371/journal.pone.0015045 (PMC2994833; doi:10.1371/journal.pone.0015045)

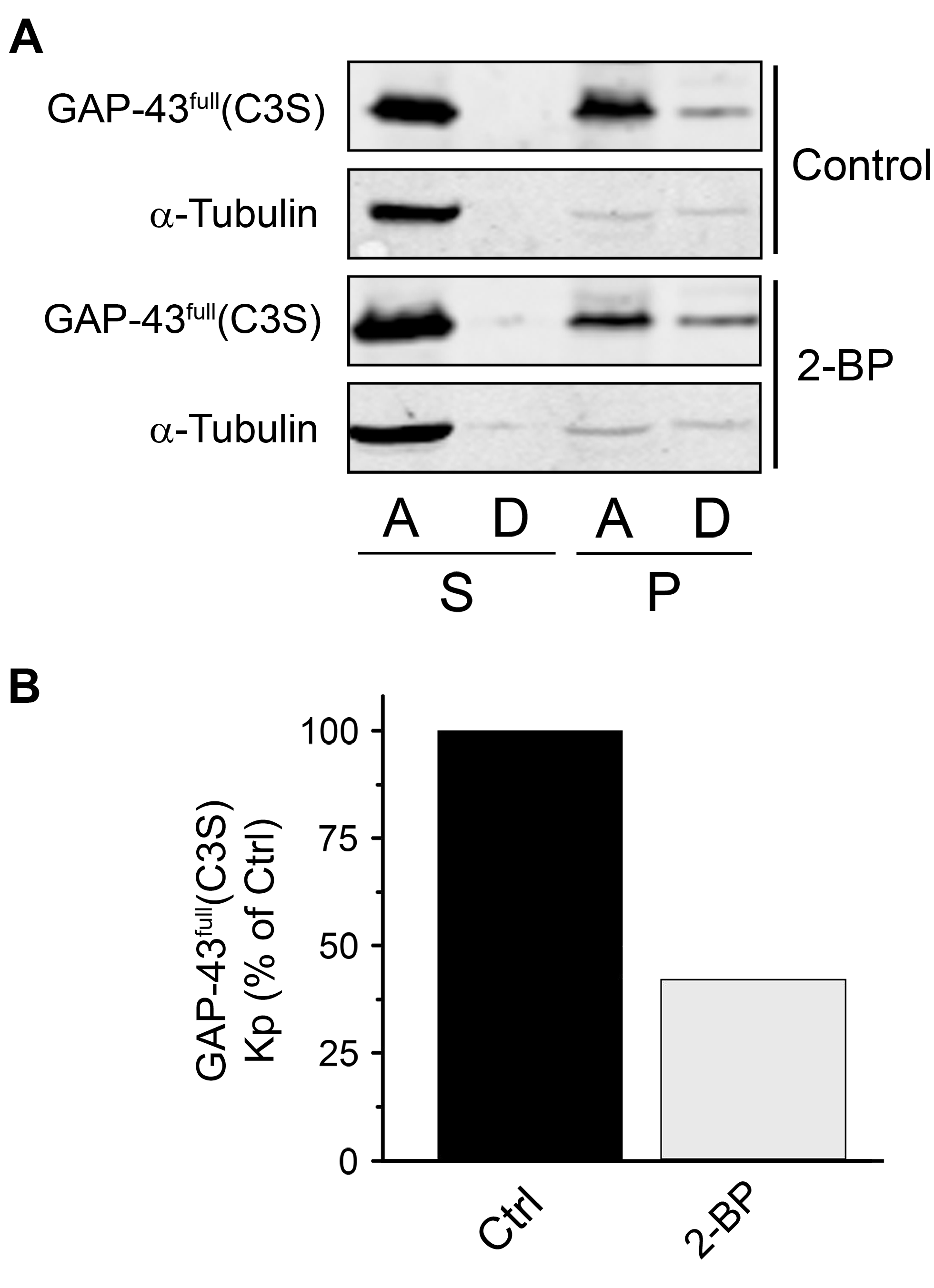

Supplement: Figure S1 — In vivo deacylation of single acylated GAP-43full(C3S). A) CHO-K1 cells transiently expressing GAP-43full(C3S)-YFP [GAP-43full(C3S)] were treated with 50 µM 2-BP (2-BP) or vehicle (Control) for 2 h. Then, cells were lysed, ultracentrifuged and the S (soluble) and P (pellet) fractions isolated. Buffer containing 1% v/v TX-114 was added to samples and the phase separation was induced at 37°C. Proteins from the A (aqueous) and D (detergent) phases were Western blotted with an antibody to GFP. The lowest panels show the Western blot using antibody to α-tubulin. B) Quantification of Western blot showed in A (see Materials and Methods). Kp = K1/K2, where K1 = P GAP-43/S GAP-43 and K2 = P tubulin/S tubulin. Kp control = 100%. (TIF) [file pone.0015045.s001.tif]

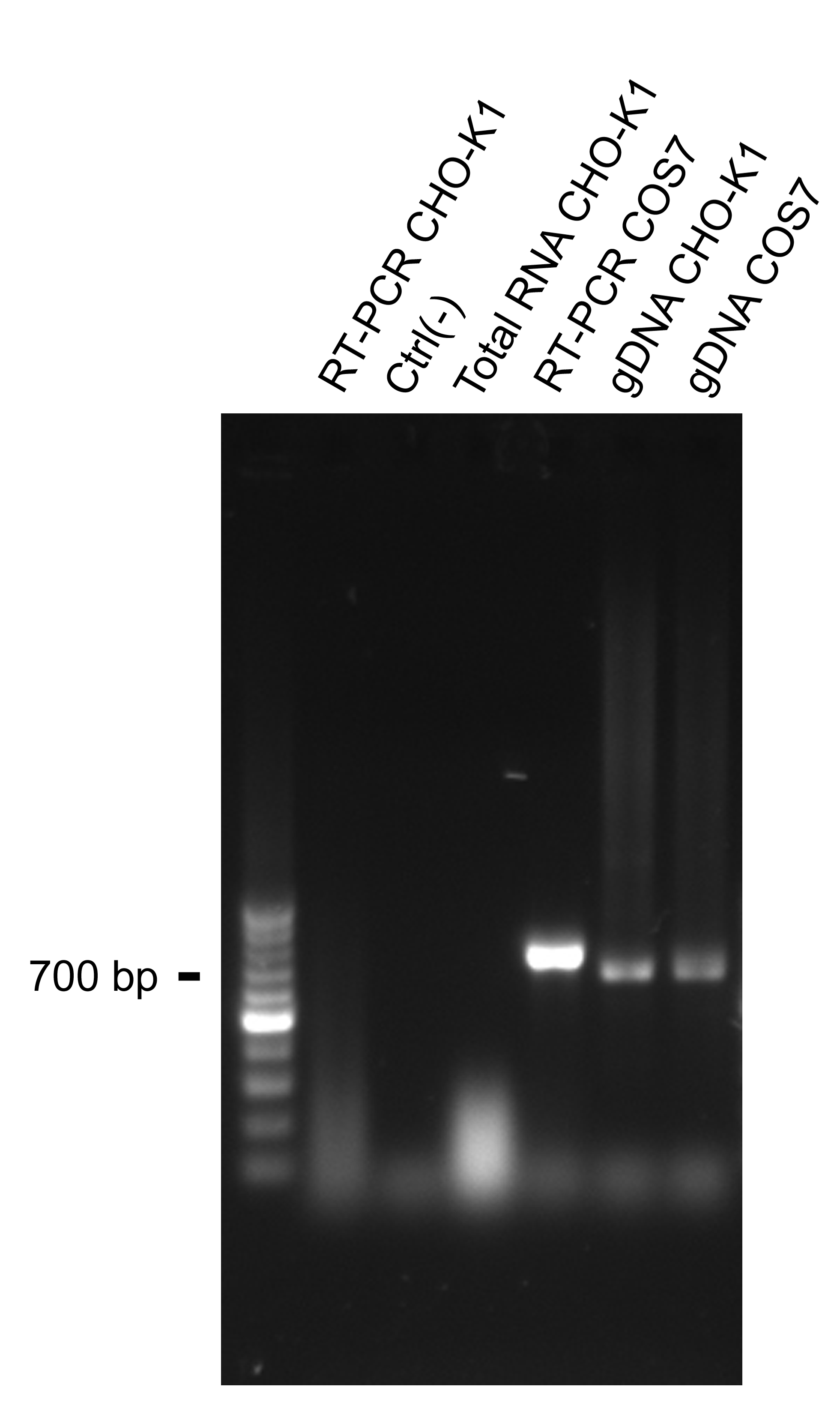

Supplement: Figure S2 — APT-1 expression analysis in COS-7 and CHO-K1 cells. PCR analysis of APT-1 expression using the following templates: first-strand cDNA obtained from RT of CHO-K1 cells purified mRNA (RT-PCR CHO-K1); the reaction mixture [Ctrl (-)]; total mRNA from CHO-K1 cells (Total RNA CHO-K1); first-strand cDNA obtained from RT of COS-7 cells purified mRNA (RT-PCR COS-7); genomic DNA from CHO-K1 cells (gDNA CHO-K1) and from COS-7 cells (gDNA COS-7). Genomic DNA and mRNA were extracted from CHO-K1 and COS-7 cells 2 day after seeding in 100 mm Petri dishes. (TIF) [file pone.0015045.s002.tif]

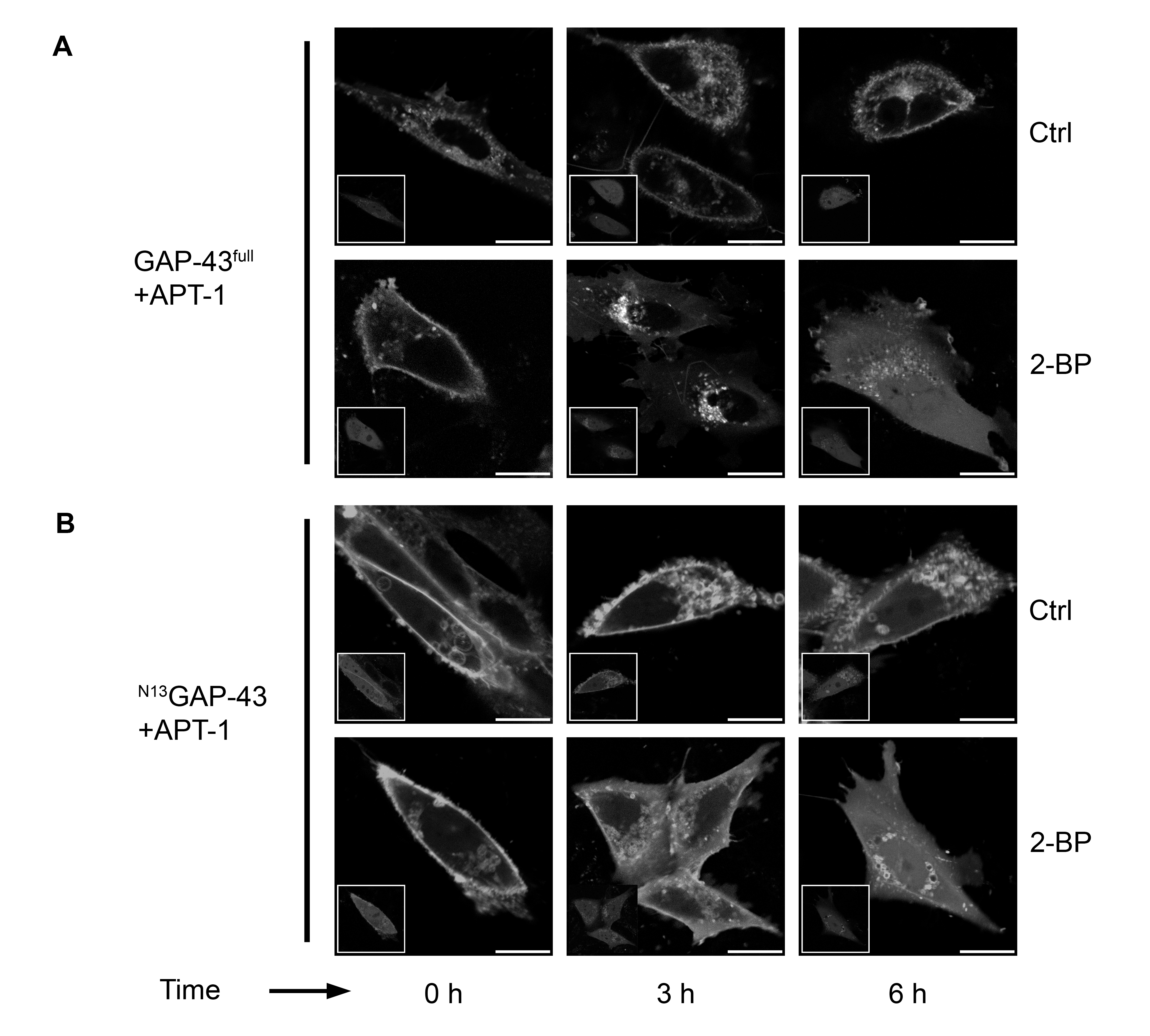

Supplement: Figure S3 — APT-1 overexpression did not significantly modify membrane association and deacylation kinetic of diacylated N13GAP-43 and GAP-43full. 60 h after transient transfection, CHO-K1 cells coexpressing GAP-43full and APT-1 (A) or N13GAP-43 and APT-1 (B) were treated with 50 µM 2-BP (2-BP) or vehicle (Ctrl) in the presence of CHX and protein degradation inhibitors for 0, 3 and 6 h and the GAP-43 subcellular distribution was analyzed by live cell confocal microscopy. Each panel shows image from YFP fluorescence (pseudocolored gray). The insets show the cells expressing APT-1 (cherry fluorescence, pseudocolored gray). Scale bar: 5 µm. (TIF) [file pone.0015045.s003.tif]

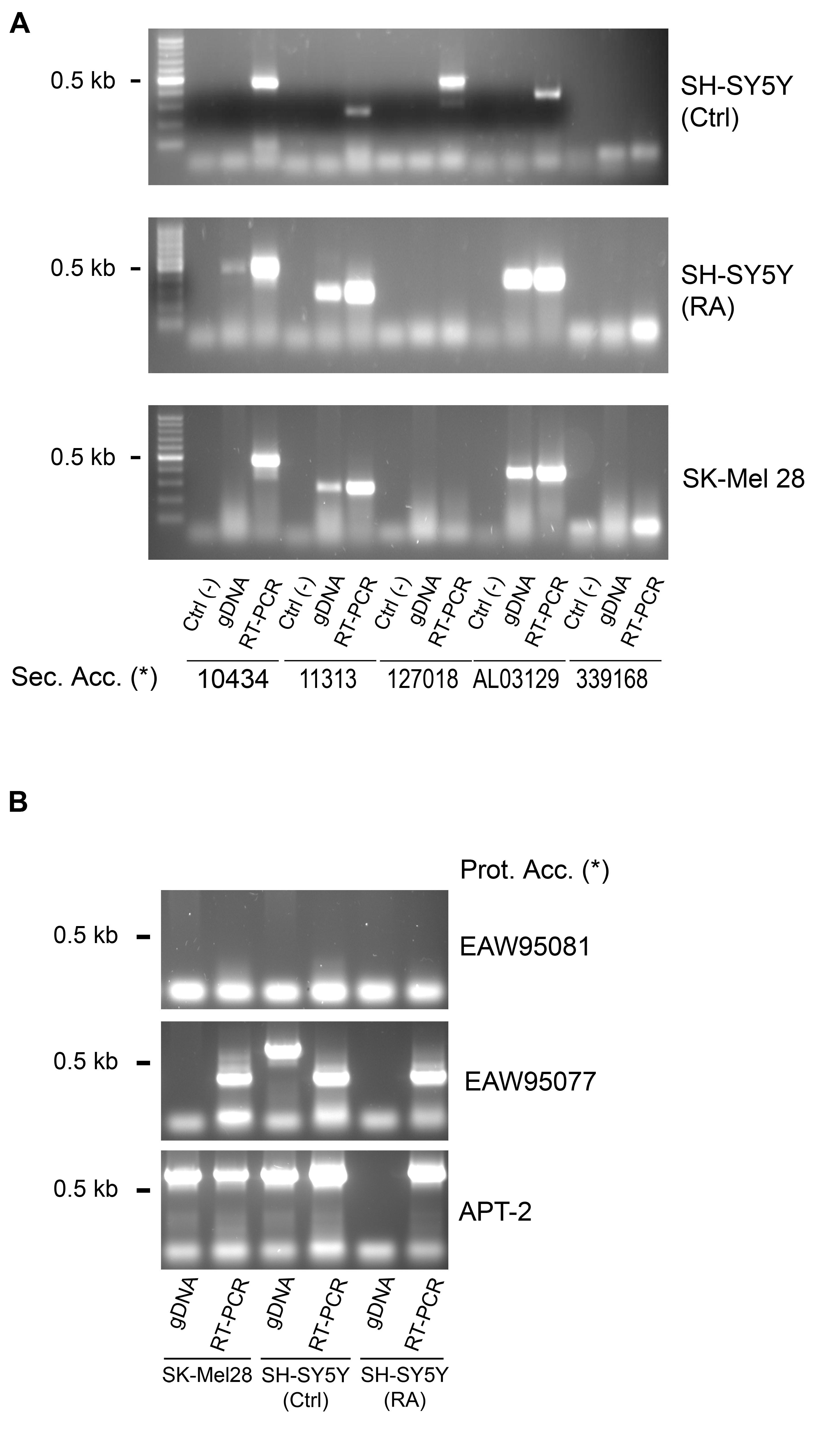

Supplement: Figure S4 — Analysis of acyl-protein thioesterase expression in SH-SY5Y and SK-Mel 28 cells. A) PCR screening of acyl-protein thioesterase gene expression in SH-SY5Y, treated (RA) or not (Ctrl) with retinoic acid (middle and upper panels, respectively), and SK-Mel 28 human cells (lower panels). For each indicated gene (see gene accession number at the bottom), PCRs were performed using specific primers and the following templates: reaction mixture [Ctrl (-)]; SH-SY5Y or SK-Mel 28 genomic DNA (gDNA) and first-strand cDNA obtained from RT of SH-SY5Y or SK-Mel 28 cells purified mRNA (RT-PCR). B) PCR analysis of isoform expression coded by gene # 11313. For each isoform indicated at the right (with the corresponding accession number), PCRs were performed using specific primers and the following templates: first-strand cDNA obtained from RT of SH-SY5Y [treated (RA) or not (Ctrl) with retinoic acid] or SK-Mel 28 cells purified mRNA (RT-PCR); SH-SY5Y or SK-Mel 28 genomic DNA (gDNA). (TIF) [file pone.0015045.s004.tif]
