# Supplementary material for: Acyl-Protein Thioesterase 2 Catalizes the Deacylation of Peripheral Membrane-Associated GAP-43
Source: PLoS One. 2010 Nov 30;5(11):e15045. doi: 10.1371/journal.pone.0015045 (PMC2994833; doi:10.1371/journal.pone.0015045)
Supplement: Table S1 — Bioinformatic data of genes potentially coding acyl-protein thioesterases and PCR primer sequences. (DOC) [file pone.0015045.s005.doc]

**Table S1. Bioinformatic data of genes potentially coding acyl-protein thioesterases and PCR primers sequences.**

| **Nucleotide sequence accesion number** | **Primers**  **[Forward (F) and Reverse (R)]** | **Isoforms**  **(Protein sequence accesión number)** | **PCR product**  **(pb)** |
| --- | --- | --- | --- |
| **Gene ID: 10434** | **5´-atgtgcggcaataacatgtcaacc-3´ (F)**  **5´-atgtgcggcaataacatgtcaacc-3´ (R)** | *NP_006321.1 (APT-1)*  AAD26994.1  EAW86743  EAW86746 | **471**  **423**  **459**  **402** |
| **Gene ID: 11313** | **5´-atgaagatggtgatgccctcctg-3´ (F)**  **5´-ctgggggaaggcccggtgca-3´ (R)** | *NP_009191 (APT-2)*  AAP97210.1  Q5QPN0  EAW95081 (iso 19)  EAW95077 (iso 23) | **281**  **273**  **267**  **264**  **225** |
| **Gene ID: 127018** | **5´-atggcggctgcgtcggggtc-3´ (F)**  **5´-ctggtaaacagcagatgctttattc-3´ (R)** | NP_620149  Q5VWZ2  BAF84231  AAQ17077  EAW93321 | **477**  **477**  **477**  **429**  **477** |
| **Nucleotide:** **AL031295** | **5´-cacagctgggctgacgccctc-3´ (F)**  **5-´ctgggggaaggcccggtgca-3´ (R)** | Q5QPQ2  Q5QPQ3  Q5QPQ1 | **357**  **357**  **282** |
| **Gene ID: 339168** | **5´-atgtggaggctggcactaggcg-3´ (F)**  **5´-gggccagcaggacacctccg-3´ (R)** | Q3KNT9  EAW90206  EAW90205  NP_937797 | **414**  **414**  **414**  **444** |
