# Supplementary material for: Acyl-Protein Thioesterase 2 Catalizes the Deacylation of Peripheral Membrane-Associated GAP-43
Source: PLoS One. 2010 Nov 30;5(11):e15045. doi: 10.1371/journal.pone.0015045 (PMC2994833; doi:10.1371/journal.pone.0015045)
Supplement: Table S2 — Aminoacid and nucleotide sequence alignments of APT-2 from CHO-K1 and Hela cells. (DOC) [file pone.0015045.s006.doc]

**Table S2. Aminoacid and nucleotide sequence alignments of APT-2 from CHO-K1 and Hela cells.**

**A) Aminoacid sequence alignments of APT-2 from CHO-K1 and Hela cells**

CHO-K1 1 MCGNTMSVPLLTDAATVSGAERETAAVIFLHGLGDTGHSWADALSTIRLPHVKYICPHAP 60

MCGNTMSVPLLTDAATVSGAERETAAVIFLHGLGDTGHSWADALSTIRLPHVKYICPHAP

HeLa 1 MCGNTMSVPLLTDAATVSGAERETAAVIFLHGLGDTGHSWADALSTIRLPHVKYICPHAP 60

CHO-K1 61 RIPVTLNMKMVMPSWFDLMGLSPDAPEDEVGIKKAAENIKALIEHEMKNGIPANRIVLGG 120

RIPVTLNMKMVMPSWFDLMGLSPDAPEDE GIKKAAENIKALIEHEMKNGIPANRIVLGG

HeLa 61 RIPVTLNMKMVMPSWFDLMGLSPDAPEDEAGIKKAAENIKALIEHEMKNGIPANRIVLGG 120

CHO-K1 121 FSQGGALSLYTALTCPHPLAGIVALSCWLPLHRNFPQAANGSAKDLAILQCHGELDPMVP 180

FSQGGALSLYTALTCPHPLAGIVALSCWLPLHR FPQAANGSAKDLAILQCHGELDPMVP

HeLa 121 FSQGGALSLYTALTCPHPLAGIVALSCWLPLHRAFPQAANGSAKDLAILQCHGELDPMVP 180

CHO-K1 181 VRFGALTAEKLRSVVTPARVQFKTYPGVMHSSCPQEMAAVKEFLEKLLPPV 231

VRFGALTAEKLRSVVTPARVQFKTYPGVMHSSCPQEMAAVKEFLEKLLPPV

HeLa 181 VRFGALTAEKLRSVVTPARVQFKTYPGVMHSSCPQEMAAVKEFLEKLLPPV 231

**Aminoacid identity: 99% (229/231)**

**B) Nucleotide sequence alignments of APT-2 from CHO-K1 and Hela cells**

CHO-K1 1 ATGTGTGGTAACACCATGTCTGTGCCCCTGCTCACTGACGCTGCCACCGTGTCTGGAGCT 60

**||||||||||||||||||||||||||||||||||| || |||||||||||||||||||||**

HeLa 1 ATGTGTGGTAACACCATGTCTGTGCCCCTGCTCACCGATGCTGCCACCGTGTCTGGAGCT 60

CHO-K1 61 GAGCGGGAAACGGCCGCGGTTATTTTTTTACATGGACTTGGAGACACAGGGCACAGCTGG 120

**||||||||||||||||||||||||||||||||||||||||||||||||||||||||||||**

HeLa 61 GAGCGGGAAACGGCCGCGGTTATTTTTTTACATGGACTTGGAGACACAGGGCACAGCTGG 120

CHO-K1 121 GCTGACGCCCTCTCCACCATCCGGCTTCCTCATGTCAAGTACATCTGTCCTCATGCGCCC 180

**|||||||||||||||||||||||||| ||||| ||||||||||||||||| ||||||||**

HeLa 121 GCTGACGCCCTCTCCACCATCCGGCTCCCTCACGTCAAGTACATCTGTCCCCATGCGCCT 180

CHO-K1 181 AGGATCCCTGTGACACTCAACATGAAGATGGTGATGCCTTCCTGGTTTGACCTGATGGGG 240

**|||||||||||||| ||||||||||||||||||||||| |||||||||||||||||||||**

HeLa 181 AGGATCCCTGTGACCCTCAACATGAAGATGGTGATGCCCTCCTGGTTTGACCTGATGGGG 240

CHO-K1 241 CTGAGTCCAGACGCCCCAGAGGATGAAGTTGGCATCAAGAAGGCAGCAGAGAACATCAAG 300

**||||||||||| ||||||||||| || | |||||||||||||||||||||||||||||||**

HeLa 241 CTGAGTCCAGATGCCCCAGAGGACGAGGCTGGCATCAAGAAGGCAGCAGAGAACATCAAG 300

CHO-K1 301 GCTTTGATTGAACATGAGATGAAGAATGGGATCCCTGCCAATCGAATCGTCCTGGGTGGC 360

**|| |||||||| ||||| |||||||| ||||||||||||||||||||||||||||| |||**

HeLa 301 GCCTTGATTGAGCATGAAATGAAGAACGGGATCCCTGCCAATCGAATCGTCCTGGGAGGC 360

CHO-K1 361 TTTTCGCAGGGCGGGGCCCTGTCCCTCTATACAGCCCTTACCTGCCCCCACCCTTTGGCT 420

**||||| ||||||||||||||||||||||| || ||||| ||||||||||||||| |||||**

HeLa 361 TTTTCACAGGGCGGGGCCCTGTCCCTCTACACGGCCCTCACCTGCCCCCACCCTCTGGCT 420

CHO-K1 421 GGCATCGTGGCATTGAGCTGTTGGCTGCCTCTGCATCGGAACTTTCCTCAAGCAGCCAAT 480

**||||||||||| |||||||| |||||||||||||| ||| ||| || || ||||| |||**

HeLa 421 GGCATCGTGGCGTTGAGCTGCTGGCTGCCTCTGCACCGGGCCTTCCCCCAGGCAGCTAAT 480

CHO-K1 481 GGTAGTGCCAAGGACCTGGCCATCCTTCAATGCCATGGGGAGCTGGATCCCATGGTACCT 540

**|| |||||||||||||||||||| || || ||||||||||||||||| |||||||| ||**

HeLa 481 GGCAGTGCCAAGGACCTGGCCATACTCCAGTGCCATGGGGAGCTGGACCCCATGGTGCCC 540

CHO-K1 541 GTTCGGTTCGGGGCCCTGACAGCTGAGAAGCTACGGTCTGTTGTCACACCTGCCAGGGTC 600

**|| ||||| ||||||||||| ||||||||||| |||||||||||||||||||||||||||**

HeLa 541 GTACGGTTTGGGGCCCTGACGGCTGAGAAGCTCCGGTCTGTTGTCACACCTGCCAGGGTC 600

CHO-K1 601 CAGTTCAAGACATACCCAGGTGTCATGCACAGCTCCTGTCCTCAGGAGATGGCAGCTGTA 660

**||||||||||||||||| |||||||||||||||||||||||||||||||||||||||||**

HeLa 601 CAGTTCAAGACATACCCGGGTGTCATGCACAGCTCCTGTCCTCAGGAGATGGCAGCTGTG 660

CHO-K1 661 AAGGAATTTCTGGAGAAGCTGCTGCCTCCTGTCTAA 696

**||||||||||| ||||||||||||||||||||||||**

HeLa 661 AAGGAATTTCTTGAGAAGCTGCTGCCTCCTGTCTAA 696

**Nucleotide identity: 93% (651/696)**
